# Phytochemicals from Cactaceae family for cancer prevention and therapy

**DOI:** 10.3389/fphar.2024.1421136

**Published:** 2024-10-24

**Authors:** Arturo Orozco-Barocio, Marina A. Sánchez-Sánchez, Argelia E. Rojas-Mayorquín, Marisol Godínez-Rubí, María Paulina Reyes-Mata, Daniel Ortuño-Sahagún

**Affiliations:** ^1^ Laboratorio de Inmunobiología, Departamento de Biología Celular y Molecular, Centro Universitario de Ciencias Biológicas y Agropecuarias, Universidad de Guadalajara, Zapopan, Mexico; ^2^ Departamento de Clínicas Médicas, Centro Universitario de Ciencias de la Salud, Universidad de Guadalajara, Guadalajara, Mexico; ^3^ Departamento de Ciencias Ambientales, Centro Universitario de Ciencias Biológicas y Agropecuarias, Universidad de Guadalajara, Zapopan, Mexico; ^4^ Laboratorio de Patología Diagnóstica e Inmunohistoquímica, Departamento de Microbiología y Patología, Centro Universitario de Ciencias de la Salud, Universidad de Guadalajara, Guadalajara, Mexico; ^5^ Departamento de Disciplinas Filosófico, Metodológicas e Instrumentales, Centro Universitario de Ciencias de la Salud, Universidad de Guadalajara, Guadalajara, Mexico; ^6^ Laboratorio de Neuroinmunobiología Molecular, Instituto de Investigación en Ciencias Biomédicas (IICB), Departamento de Biología Molecular y Genómica, Universidad de Guadalajara, Centro Universitario de Ciencias de la Salud, Guadalajara Mexico

**Keywords:** anticancerigens, tradicional medicine, Cactacea, phytochemical, antitumor

## Abstract

Cancer is a global health issue, increasingly prevalent and a leading cause of mortality. Despite extensive research, conventional treatments remain aggressive, often damaging healthy cells, and exhibit limited efficacy. Addressing drug resistance and enhancing treatment effectiveness are critical challenges in advancing cancer therapy. This review examines the potential of natural plant compounds, particularly phytochemicals and their derivatives, in developing novel anticancer agents. These metabolites have a long history in traditional medicine, with 42% of molecules approved for cancer treatment between 1981 and 2019 being either natural products or derivatives. The Cactaceae family, which comprises more than 1,500 species, represents a largely untapped source of potentially useful chemopreventive and anticancer agents. Although more than 3,000 plants and their derivatives have contributed to chemotherapeutic development, cactus species have received limited attention until recently. Emerging evidence highlights the anticancer potential of fruits, stems, and cladodes from various cactus species. This review provides a comprehensive and current overview of experimental studies on Cactaceae in cancer research, aiming to pave the way for the development of innovative, natural cancer therapeutics and contribute to the ongoing battle against this formidable disease.

## 1 Introduction

Non-communicable diseases presently constitute the predominant cause of global mortality, with cancer anticipated to emerge as the primary cause of death and a significant impediment to increased life expectancy across all nations in the 21st century (Bray et al., 2024). The World Health Organization (WHO) projects cancer as either the first or second leading cause of premature mortality, under the age of 70, in 177 out of 183 countries, and the third or fourth leading cause of death in a further 23 countries (World Health Organization, 2024). Consequently, cancer is escalating worldwide, and is one of the main causes of mortality, with an estimated 35 million new cases forecasted for 2050, signifying a 77% surge from 2022 levels (Bray et al., 2024).

Chemotherapy remains a cornerstone of cancer treatment and requires the use of different chemotherapeutic agents targeting different cancer properties and pathways (Iqbal et al., 2017). Nonetheless, the emergence of multidrug resistance represents a significant obstacle to successful cancer therapy and the containment of metastasis (Costea et al., 2020). Mechanisms of multidrug resistance include increased drug efflux, drug inactivation, detoxification pathways, alteration of target structures, inhibition of apoptosis, involvement of cancer stem cells, miRNA dysregulation, epigenetic modifications, DNA repair defects, tumor heterogeneity, microenvironmental influences, epithelial-mesenchymal transition and modulation of reactive oxygen species (Bukowski et al., 2020). To improve the efficacy of cancer treatment, agents must be developed that are able to circumvent the mechanisms of drug resistance. To this end, researchers are investigating the potential of natural products in combating various facets of drug resistance (Keyvani-Ghamsari et al., 2020).

Phytochemicals and their derivatives, obtained from plant sources, have long been employed in traditional medicine practices (Rizeq et al., 2020). These compounds, rich in beneficial nutrients and biologically active metabolites, have garnered significant interest for their potential roles in preventing and treating a myriad of ailments, including hypercholesterolemia, diabetes, cardiovascular disease, hypoglycemia, hypolipidemia, edema, joint pain, weight control, visual disorders, neuroprotective effects, and asthma (Shoukat et al., 2023). With an increasing focus on alternative botanical medicines, there is growing recognition of their potential in developing anticancer therapies with reduced side effects (Dey et al., 2019; Majolo et al., 2019; Choudhari et al., 2020; Khan et al., 2019). Notably, a substantial proportion of cancer treatments (around 42%) approved between 1981 and 2019 were either natural products or their derivatives, highlighting their significance in drug development. Significantly, 114 of the 247 anticancer molecules released (46%) during the same period were derived from natural products and their derivatives (Newman and Cragg, 2020). Despite the extensive diversity within the cactus family, comprising over 1,500 species, only a limited number have been investigated for their chemopreventive and anticancer properties, presenting an untapped avenue for further exploration (Harlev et al., 2013; Allegra et al., 2019).

The term “cactus” covers a diverse collection of succulent species, comprising about 130 genera and 1,500 species within the family Cactaceae (order *Caryophyllales*). As the second largest family in the Neotropics, the Cactaceae exhibit a broad spectrum of morphologies and sizes, characterized by rapid growth, adaptation to nutrient-poor soils and low water requirements (da Silva-Santos, et al., 2021; Santos Díaz et al., 2017; Bravo-Hollis and Sánchez-Mejorada, 1978). The extensive diversity of the Cactaceae is notably documented in regions such as Mexico, which harbors 586 species, as well as in the southwestern United States of America, the central Andes (including Peru, Bolivia, southern Ecuador, northeastern Chile, and northwestern Argentina), Brazil, Paraguay, Uruguay, and Argentina (Das et al., 2021; Santos Díaz et al., 2017; Bravo-Hollis and Sánchez-Mejorada, 1978).

Cactaceae, a remarkable plant family has evolved fascinating adaptations to thrive in arid and semi-arid regions across the world. The Cactaceae family includes two main subfamilies: *Cactoideae and Opuntioideae*. *Cactoideae* represent the largest and most diverse subfamily containing specialized succulents and includes several genera such as *Hylocereus*, *Cereus*, *Pilosocereus*, *Melocactus*, *Stenocereus, Cephalocereus*, *Myrtillocactus, Lophophora*, and *Lophocereus.* The subfamily Opuntioideae, which comprise leafless, stem-succulent plants with numerous spines, includes two small genera, *Pereskia* (Pereskioideae) and *Maihuenia* (Maihuenioideae) (da Silveira Agostini-Costa, 2020; Santos Díaz et al., 2017; Bravo Hollis and Sánchez-Mejorada, 1991). Cacti are widely used and exploited in different cultures around the world: as food (bread and cakes, juice, ice cream, yogurt, gummy candies, refreshing and alcoholic beverages), cosmetics, even as ornamental plants, as fodder for ruminants, hedges, living fences, firewood, as construction material, and in folk medicine (Monteiro, et al., 2023; Shoukat, et al., 2023; Martins, et al., 2023; García-Cruz, et al., 2022; De Araujo, et al., 2021; da Silva Santos, et al., 2021; Das et al., 2021). Traditionally, people find utility in various cacti parts. Primarily, the cladodes, stem, fruits, and roots are used. Subsequently, the flowers, mucilages, and seeds also serve for practical purposes. (da Silveira Agostini-Costa, 2020; Das et al., 2021; Bravo Hollis and Sánchez-Mejorada, 1991).

Diverse studies have confirmed the traditional use of cactus species against several disorders, such as: metabolic disorders, as a blood sugar lowering, diabetes mellitus, obesity, hypertension, antiatherogenic, antihyperlipidemic, antihypercholesterolemic, antioxidant and diuretic, cardioprotective, hepatoprotective, wound disinfectant, dysentery treatment, adjuvant in venomous snake bites, elimination of parasites, antimicrobial, anti-inflammatory, analgesic, wound healing, anti-ulcer, anti-proliferative properties, and anti-cancer (Shoukat, et al., 2023; Martins, et al., 2023; Madrigal-Santillán et al., 2022; García-Cruz, et al., 2022; De Araujo, et al., 2021; da Silveira Agostini-Costa, 2020; Ramírez-Rodríguez, et al., 2020; Harlev, et al., 2013). In a recent review, Das et al. show a list of 42 cactus species ethnobotanically used as botanical medicines and the parts of the cactus used (Das et al., 2021).

Cactus plants synthesize diverse chemical metabolites in response to various environmental stimuli, encompassing polyphenols, alkaloids, betalains, terpenes, and fatty acids, with nutritional, pharmacological, and food-related applications (Das et al., 2021; Harlev et al., 2013; Gandía-Herrero et al., 2016; Santos Díaz et al., 2017; da Silveira Agostini-Costa, 2020; Madrigal-Santillán et al., 2022). Scientific literature has elucidated the therapeutic potential of cactus species in combating degenerative diseases, notably cancer, supported by extensive research efforts (Harlev et al., 2013; Gandía-Herrero et al., 2016; Santos Díaz et al., 2017; Iqbal et al., 2017; da Silveira Agostini-Costa, 2020; Madrigal-Santillán et al., 2022; Shoukat et al., 2023; Martins et al., 2023). Moreover, numerous chemotherapeutic agents utilized in cancer treatment derive from plant sources, with carotenoids, terpenoids, and alkaloids among the primary metabolites (Shoukat et al., 2023). Actually, cactus fruits, stems, and cladodes have garnered significant attention for their anticancer properties, supported by international reports and emerging scientific evidence (Das et al., 2021; Daniloski et al., 2022; Madrigal-Santillán et al., 2022; De Araujo et al., 2021; da Silveira Agostini-Costa, 2020).

This work aimed to provide an up-to-date and comprehensive review of the experimental work carried out to date with plants of the Cactaceae family in cancer research. A literature search was performed in the PubMed, Web of Science, Scopus, and Science Direct databases. The keywords and phrases used in the search were “medicinal plants AND cancer”, “plant extract AND anti-cancer”, “Cactaceae AND cancer” and “cactus AND cancer”. The species that were included after analyzing the above keywords were those that reported the traditional use of the plant or its parts against cancer and those that reported anticancer effects of the plant extracts or metabolites in both *in vivo* and *in vitro* models. Care was taken to ensure that only cactus species whose taxonomic identification data were registered in an academic institution with an identification document were included. Care was also taken to ensure that all research articles were published in peer-reviewed journals. Likewise, the intention of this review is to compile the information known so far about cacti in cancer treatment, to present the scientific methods used and the phytochemicals identified in order to stimulate further research on the possible mechanisms of action of these plants in the biology of cancer and the influence on immunological mechanisms.

## 2 Cactaceae genus with antitumor effects of extracts and metabolites derived from

**FIGURE 1:**
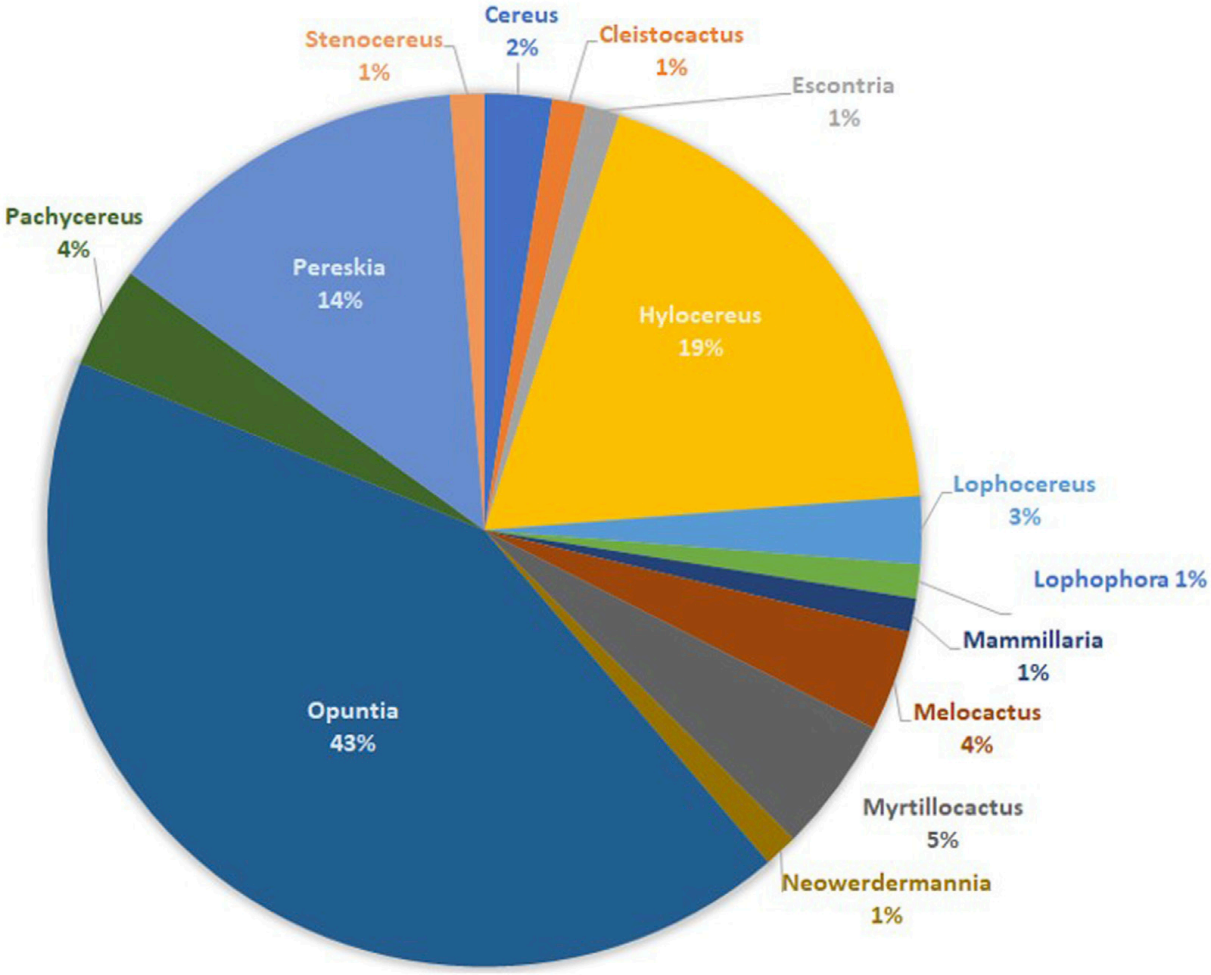
The most important Cactaceae genera, analyzed according to the percentage of original references (N = 80).

**FIGURE 2:**
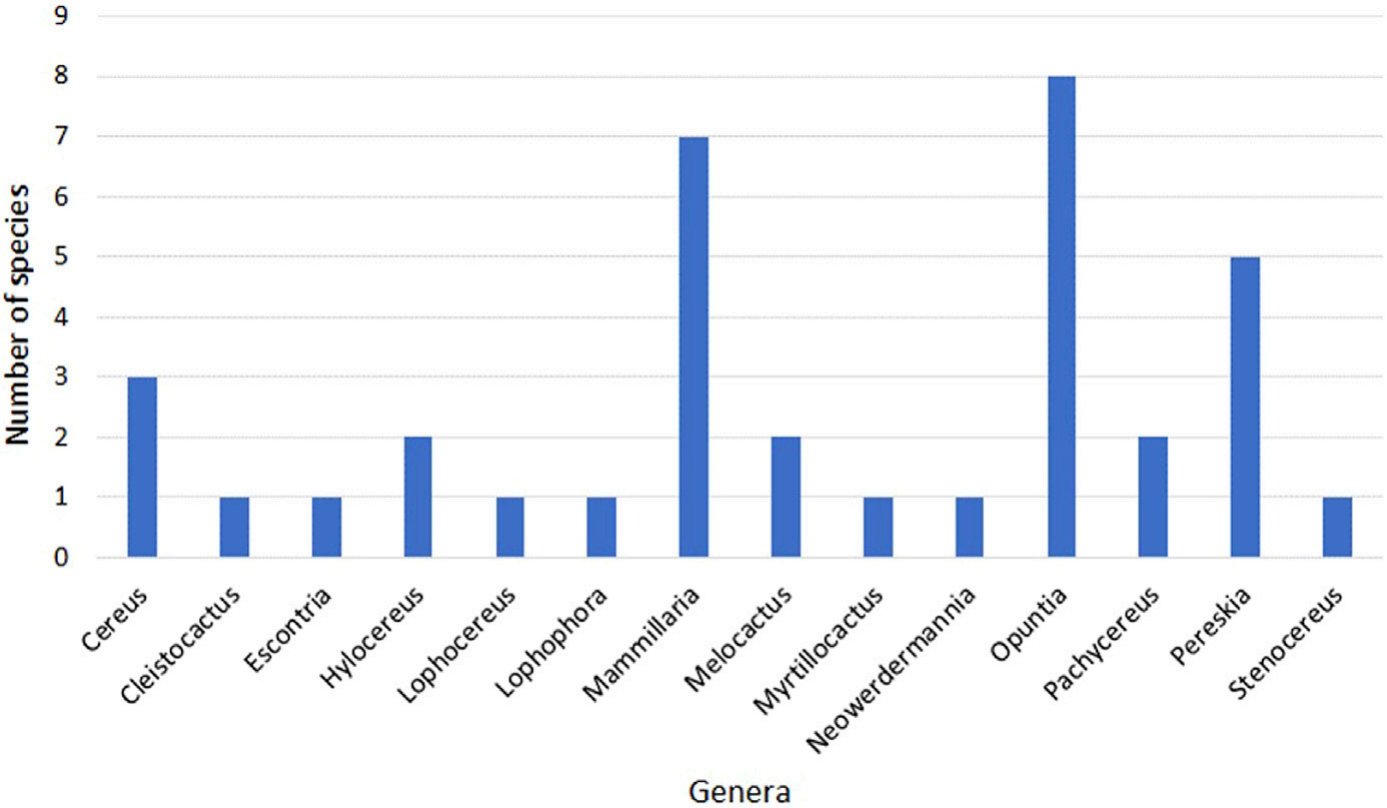
Number of different species by genus investigated in anticancer studies.

After an extensive search and selection of references reporting anti-cancer effects of extracts from various Cactaceae plants, we compile in this review the information from eighty original references from 14 genera of the Cactaceae family (Figure 1). Three genera have been studied the most so far: *Opuntia*, *Hylocereus* and *Pereskia*, accounting for about 76% of the studies. However, if we analyze the different species included in the studies for each genus (Figure 2), we find that two of the three genera with the most studied species are again *Opuntia* (with eight studied species) and *Pereskia* (with five studied species) and also including *Mammillaria* (with six studied species).

### 2.1 Genus *Cereus*

**FIGURE 3:**
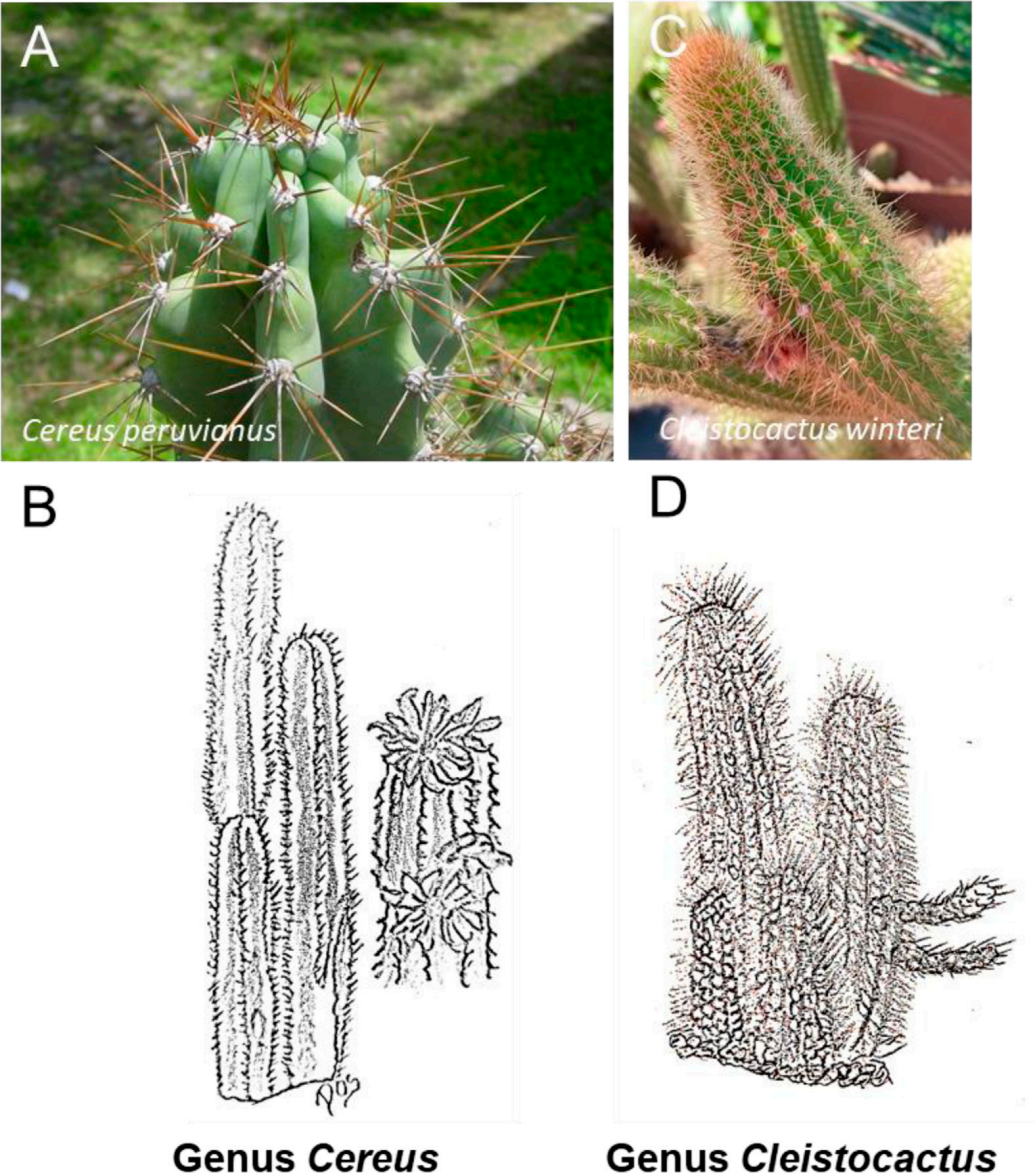
Schematic representative draw **(B, D)** and detailed picture **(A, C)** for the genus *Cereus* **(A, B)** and *Cleistocactus* **(C, D)** of cactaceae family. The most common representative of the Cactaceae from the genus *Cereus* is the *Cereus peruvianus*, also known as the Peruvian apple cactus or the Queen of the Night. From the genus *Cleistocactus* one of the most common representatives is the *Cleistocactus strausii*, also known as the “Silver Torch” or “Wooly Torch.”

*Cereus* species are characterized as large, tree-like, columnar cacti exhibiting four to ten pronounced ribs, thick stems, large white flowers and delicious pink or red fruits that emerge from the edges of the ribs. These plants show remarkable resilience to temperatures from −5°C to 45°C and exhibit tolerance to various soil compositions. Their natural habitat spans the southwestern United States of America, Mexico, Central America, and Brazil, comprising numerous species within the *Cereus* family (De Araujo, et al., 2021; Harlev, et al., 2013; da Silva-Santos, et al., 2021) (Figure 3B). Notably, *Cereus* cacti have garnered attention as potential crop plants and are also recognized for their medicinal properties, particularly in the treatment of breast cancer, with emphasis on *Cereus quadrangularis* Haw. taking center stage (Harlev, et al., 2013). In addition, extracts from *Cereus hildmannianus* cladodes are used in folk medicine for diverse purposes such as weight loss, cholesterol reduction, diuretic effect, treatment of pulmonary disorders, rheumatism, wound healing, and lithiasis management (da Silva-Santos, et al., 2021). *Cereus jamacaru* DC, commonly known as mandacaru, has been investigated for its anti-cancer and anti-tumor potential. It has been shown to reduce the viability of sarcomas, inducing a tumor reduction of 86.07%, and mitigating the cytotoxic effects of cisplatin without inducing mutagenic or cytotoxic damage in murine blood cells and human lymphocytes (De Araujo, et al., 2021; Vencioneck Dutra, et al., 2018) (Supplementary Table S1). Additionally, studies by Jacomini et al. (2015) have shown the importance of *Cereus peruvianus* Mill. (Figure 3A). callus cultures as a source of polyunsaturated fatty acids (PUFAs) and their remarkable antiproliferative activity. The raw extract inhibited the growth of Caco-2 cells (colon adenocarcinoma cells) and showed an antiproliferative effect with a CC50 value of 250 μg/mL (Jacomini, et al., 2015) (Supplementary Table S1).

### 2.2 Genus *Cleistocactus*

The genus *Cleistocactus*, which belongs to the family Cactaceae, comprises flowering plants that are indigenous to the mountainous regions of South America, particularly Peru, Uruguay, Bolivia and Argentina, at altitudes of up to 3,000 m above sea level. The etymology of the genus name comes from the Greek term “kleistos”, “ which means “closed” and is attributed to the characteristic of its flowers scarcely opening. These cacti typically have tall, slender stems, which often branching extensively with numerous ribs, closely spaced areoles, and spines. The flowers are tubular, with tips that seldomly open, typically revealing only the protrusion of the style and stamens (Lowry, 2016) (Figure 3D). Notably, prior to Mina et al.'s study in 2020, there was scant information regarding the biological activity or phytochemical composition of the genus *Cleistocactus*. Mina and colleagues assessed the cytotoxic potential of the crude alkaloid fraction extracted from *Cleistocactus winteri*, commonly known as the golden rat tail, against three human cancer cell lines: hepatocarcinoma (HepG2), breast adenocarcinoma (MCF-7), and colon adenocarcinoma (Caco-2). Their investigation revealed significant cytotoxic effects, with IC50 values of 26.53 μg/mL, 23.8 μg/mL, and 13.07 μg/mL, respectively. Notably, these values demonstrate nearly equivalent cytotoxic activity to that of Doxorubicin, employed as a positive control in the study (Mina et al., 2020) (Figure 3C) (Supplementary Table S1). These results could stimulate further research on the cytotoxic properties of the extracts from this Cactaceae family.

### 2.3 Genus *Escontrias*

**FIGURE 4:**
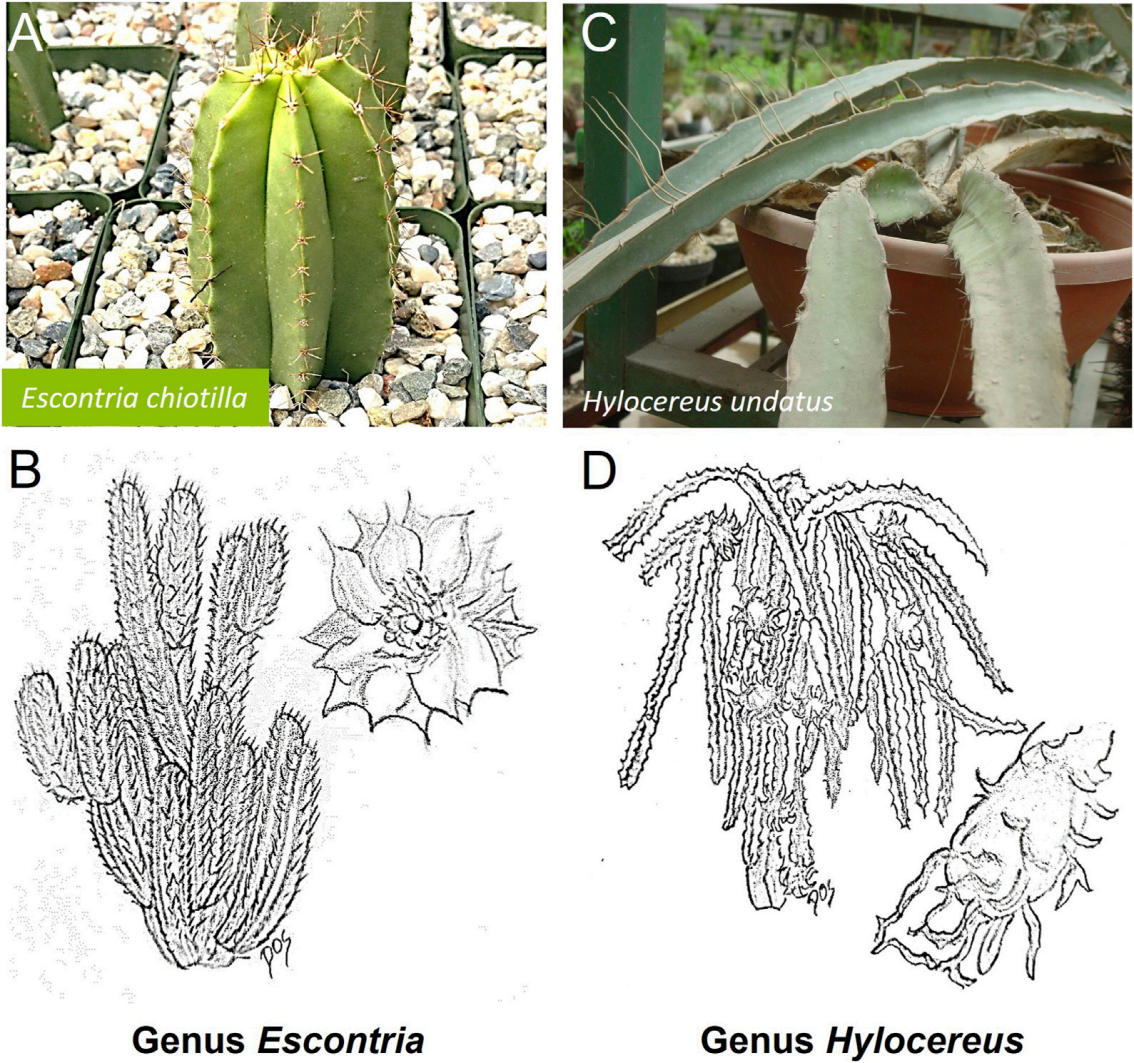
Schematic representative draw **(B, D)** and detailed picture **(A, C)** for the genus *Escontria* **(A, B)** and *Hylocereus* **(C, D)** of cactaceae family. The most common representative of the Cactaceae from the genus *Escontria* is *Escontria chiotilla*, commonly referred to as “Chiotilla” or “Chichituna.”. From the genus *Hylocereus* is *Hylocereus undatus*, known as the “Dragon Fruit” or “Pitaya.”

*Escontria chiotilla*, colloquially known as jiotilla, is an arboreal cactus species indigenous to southern Mexico specifically in the dryland of the states of Michoacán, Guerrero, Puebla, and Oaxaca (Bravo-Hollis and Sánchez-Mejorada, 1978) and characterized by numerous branching stems. Notably, its small flowers possess a unique “gold leaf” appearance with campanulate morphology and receptacle tubes, showcasing a metallic sheen attributed to translucent, membrane-like bracts. These cup-shaped, yellow flowers bloom twice annually, from February to May and from July to September. The fruits, resembling large grapes, exhibit purple-brown or purple-red hues in both peel and pulp, featuring a bittersweet flavor. Papery bracts encase the fruit, which harbors ovoid-shaped, black seeds measuring 1–1.5 mm in length and 0.8–1.0 mm in diameter. Harvested primarily from April to May and from September to November, these fruits, marketed under different names such as “jiotilla”, “chiotilla” or “quiotilla” hold significant cultural and economic value in the region (Bravo-Hollis and Sánchez-Mejorada, 1978; Cruz-Zamora, et al., 2017) (Figures 4A–C). The fruits of *Escontria chiotilla* are consumed both fresh and in processed forms such as jam, ice cream and alcoholic beverages, distinguishing their flavor from that of other cactus species (Ramírez-Rodríguez, et al., 2020).

To date, only one study has investigated the cytotoxic activity of the clarified fruit juice of *E. chiotilla* in four different human adenocarcinoma cell lines (Sandate-Flores, et al., 2020) (Supplementary Table S1). No cytotoxicity was observed in the normal cell control (NIH/3T3 cell line), and the MCF-7 breast cancer cell lines and the Caco-2 colon cell lines also showed no cytotoxicity. The fruit juice of *E. chiotilla* showed a higher level of cytotoxicity only in the liver cancer cell line HepG2 and decreased cell viability in the prostate cancer cell line PC-3. Therefore, further research is needed to investigate this selective activity.

### 2.4 Genus *Hylocereus*

*Hylocereus*, commonly referred to as white-fleshed pitahaya or dragon fruit, belongs to the epiphytic cactus species in the Cactaceae family and is the most widely cultivated within its genus. The plant features climbing, jointed, and fleshy stems with triangular shapes consisting of three undulating wings with horny scalloped margins, reaching lengths of up to 6 m. Its nocturnal flowering produces large, fragrant bell-shaped flowers pollinated by bats and moths, measuring 30 cm long and 23 cm wide, adorned with a green, lobed stigma. The ellipsoidal pitahaya fruit, 10–12 cm in diameter and 11–30 cm in length, showcases a bittersweet flavor and vibrant colors, with bracts covering the outer skin and juicy, fleshy pulp containing evenly distributed small, shiny seeds (Verona-Ruiz, et al., 2020; Bravo Hollis and Sánchez-Mejorada, 1991). Taxonomically, the *Hylocereus* genus comprising about 17 species, with nine registered in Mexico, and is native to tropical regions of Central and South America, thriving in diverse climates and soils worldwide (Ramirez-Rodríguez, et al., 2020; De Araujo, et al., 2021) (Figure 4B). Cultivated varieties, distinguished by skin and flesh color, include *H. undatus* with red skin and white flesh (Figure 4C), *Hylocereus polyrhizus* with red skin and red flesh, and *Hylocereus megalanthus* with yellow skin and white flesh. Both fruits and flowers contain pharmacologically active compounds, making *Hylocereus undatus* a captivating cactus species prized for its ornamental appeal and culinary delights (Joshi and Prabhakar, 2020) Figure 4D.

In 2006 Wu et al., compared the antiproliferative activity of dragon fruit (pitahaya) peel and flesh on B16F10 melanoma cells, being better the effect of the fruit peel. The inhibition of the proliferation was dose-dependent in relation to a higher content of flavonoids and betalains (Wu, et al., 2006) (Supplementary Table S1). Later on, several others have reproduced the results in other cancer cell lines, by using extracts from different *Hylocereus* species, and going deep into the identification of the active metabolites and the mechanisms of the effects. Yellow piraya extract (*H. undatus*) has showed a high inhibitory effect on breast cancer MCF-7 cell proliferation (Jayakumar and Kanthimathi, 2011) (Supplementary Table S1). Also, extracts of peels of dragon fruit from *H. undatus* and *H. polyrhizus*, which contained 3 to 5 times of higher flavonoid and polyphenol content compared to fruit flesh, showed good antiproliferative activity against cancer cells of human gastric adenocarcinoma cell line (AGS) and human breast adenocarcinoma cell line (MCF-7) (Kim, et al., 2011) (Supplementary Table S1). In addition, dragon fruit peel extract presents a cytotoxic effect against PC3 (human prostate cancer cell line), Bcap-37 (human breast cancer cell line) and MGC-803 (human gastric cancer cell line) in a dose-dependent manner. The IC50 value ranged from 0.61 to 0.73 mg/ml. *Hylocereus polyrhizus* showed a better cytotoxic effect than *H. undatus* on MGC-803 cells (Luo, et al., 2014) (Supplementary Table S1).

In addition to its antiproliferative effects, different extracts from the pulp and the peel of the dragon fruit exhibits proapoptotic effects. An hydroalcoholic extract (50:50 ethanol-water) from the pulp of fruit pitahaya from *H. polyrhizus* induces a decrease in cell proliferation in MCF-7 (ER+) cell line, in which a cell cycle analysis revealed an increase in G0/G1 phase followed by a decrease in G2/M phase, but also an induction of apoptosis, by suppressing BRCA1, BRCA2, PRAB, and Erα gene expression. Interestingly, MDA-MB-435 cells (ER−) were not affected by the extract (Guimarães, et al., 2017). In another study using *H. undatus* pulp and peel extracts and determining by using Liquid Chromatography Mass Spectrometry (LC-MS) and Gas Chromatography–Mass Spectrometry (GC-MS) the main metabolites of the extracts, the total phenolic and flavonoid contents in the peel extract were significantly higher than those in the pulp extract. Although, both extracts displayed cytotoxic activity against MCF-7 and Caco-2 cancer cells after 48 h of treatment at IC50 values ranging from 14 to 53 μg/mL with high selective indices against normal WI-38 and MCF-10A cell lines. In addition, the apoptosis potential of the anticancer effects was also evaluated. In this case, the increase in apoptosis was revealed by the overexpression of p53, BAX, and caspase-9 and the downregulation of antiapoptotic Bcl-2 mRNA and protein expressions (Salam, et al., 2022) (Supplementary Table S1).

When aqueous and methanolic extracts from the pulp of pitahaya (*Hylocereus costaricensis, H. undatus, H. megalanthus*) were compared with different human cancer cell lines, moderate cytotoxic activity against cancer cells was observed, with no detectable effects on normal cells. Methanol extracts predominated, except for Caco-2 cells, where the aqueous extract at 500 μg/mL showed the highest cytotoxic activity. Methanol extracts at the same concentration showed optimal results against HT29 colon, HepG2 liver and DU145 prostate cancer cells, while the effects on normal prostate epithelial cells remained negligible (Pasko, et al., 2021a) (Supplementary Table S1). Subsequent studies extended these findings and investigated the cytotoxic activity of dragon fruit extracts on additional human cancer cell lines derived from different tissues and exhibiting different metastatic properties (including skin, prostate and gastrointestinal cancer cell lines). In addition, the antioxidant and anti-inflammatory activities of the extracts were investigated. The extracts showed significant effects on the viability of cancer cells, especially in colon and prostate cancer cells. In particular, the cytotoxic activity against colon cancer cells Caco-2 and HT-29 was significantly higher for the water extracts than for the methanol extracts, both for DI and DT. Conversely, prostate cancer cells DU145 showed higher activity with the methanol extracts than with the water extracts, with DI exerting a stronger effect on cell viability than DT. In addition, the cytotoxic effect of water and methanol extracts from DT fruit on high-grade metastatic prostate cancer cells (PC3) was significantly stronger compared to DI (Pasko, et al., 2021b) (Supplementary Table S1).

More recently, Shyamalagowri et al. (2022) prepared silver nanoparticles (AgNPs) using the aqueous extract of dragon fruit (H. undatus) peel and investigated their cytotoxic activity *in vitro*. They observed a significant cytotoxic effect of DFPAE-AgNPs against HepG2 cell lines with extensive cell death after 24 h at different concentrations (10–50 μg mL) (Shyamalagowri, et al., 2022) (Supplementary Table S1). However, when the medicinal value of natural dyes extracted from two species (*H. polyrhizus* and *H. undatus*) of dragon fruit was investigated on Ehrlich ascites carcinoma (EAC) cell line, neither species showed any remarkable cytotoxic effect on EAC cell viability at different concentrations (25, 50, 100, 200 and 400 μg/mL) (Ashaduzzaman Nur, et al., 2023) (Supplementary Table S1). El-Nashar et al. (2024) also identified flavonoids, phenolic acids, anthocyanin glycosides, lignans, stilbenes and coumarins in peel, pulp and seed extracts of white piraya (*H. undatus*) by HPLC-ESI/MS-MS. In addition, the synergistic cytotoxic potential with cisplatin against cervical cancer cells was investigated to increase efficacy and optimize treatment outcomes. In addition, the cytotoxic effect was investigated by MTT assay against prostate (PC3), breast (MDA-MB-231) and cervical cancer (HeLa) as well as against normal Vero cell lines. The seed and peel extracts showed a remarkable cytotoxic effect against all tested cell lines. They also showed downregulation of Bcl-2 and overexpression of P53 and BAX in HeLa cells treated with Pitaya extracts, which eventually activated the apoptosis process (El-Nashar, et al., 2024). Consequently, much more research is needed to clearly determine the nature of the extracts and the phytochemical metabolites that induce the cytotoxic and proapoptotic properties.

### 2.5 Genus *Lophocereus*

**FIGURE 5:**
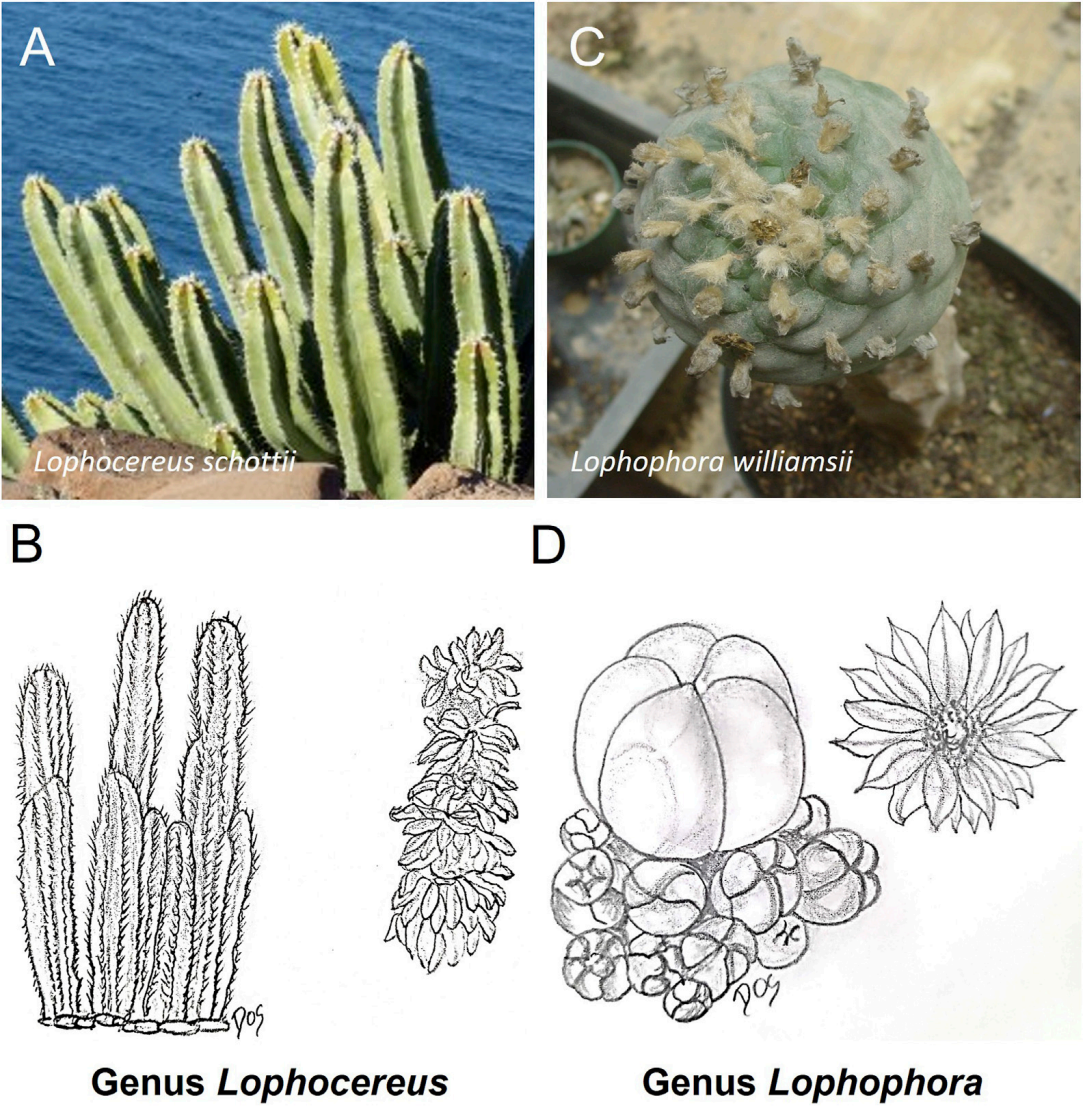
Schematic representative draw **(B, D)** and detailed picture **(A, C)** for the genus *Lophocereus* **(A, B)** and *Lophophora* **(C, D)** of cactaceae family. The most common representative of the Cactaceae from the genus *Lophocereus* is *Lophocereus schottii*, commonly known as the “senita” or “old man cactus”. From the genus *Lophophora* is *Lophophora williamsii*, commonly known as “peyote.”

*Lophocereus schottii* (Engelm) Britton & Rose, is a species of cactus endemic to Mexico. It thrives in warm climates and is typically associated with coastal dunes and xerophyte shrublands. This cactus species is characterized by 5–10 ribs and reaches a height of 2–4 m. Colloquial names include cardona, muso, sina, sinita, cactus star, mochi and viejo (meaning “old man”) (Figure 5B). Traditional medicinal practices include the use of decoctions made from the stems of *L. schottii.* (Figure 5A). These preparations are employed for treating various diseases, including cancer, diabetes, ulcers, sores, stomach disorders and tuberculosis. Notably the stems possessing five ribs are preferred for these therapeutic purposes. Chemical investigations have revealed the presence of alkaloids such as lophocerine and pilocerine in this species (Bravo-Hollis and Sánchez-Mejorada, 1978; Argueta, et al., 1994; Djerassi, et al., 1958; Shetty, et al., 2012; da Silveira Agostini-Costa, 2020). These metabolites contribute to its pharmacological properties. In laboratory studies, we explored the effects of an ethanolic extract derived from the stems of *L. schottii* on murine L5178Y lymphoma. Following oral, intratumoral and intramuscular administration to inoculated mice, this extract resulted in prolonged survival and a reduction in tumor mass (Orozco-Barocio et al., 2013) (Table 1). Recently, our studies have highlighted lophocerine as a promising anticarcinogenic phytochemical. *In vitro* experiments demonstrated that both the ethanolic extract and its polar fraction (PFLs) from *L. schottii* exhibited remarkable antiproliferative activity. In particular, the polar fraction showed higher efficacy. It inhibited cell viability in stimulated splenocytes and L5178Y cells significantly more than in non-stimulated cases. UPLC-MS analyzes revealed that the polar fraction, which was enriched with 86% lophocerine, contained the most potent anticarcinogenic metabolites (Orozco-Barocio et al., 2022) (Supplementary Table S1).

**TABLE 1:** In vivo evidence for anticancer properties of metabolites from cacti. Arranged alphabetically.

Genus/specie	Part of cactus	Extract type	Metabolites	In vivo model	Dosis / Effect observed	Molecular / cellular effect	Reference
*Lophocereus schottii*	Stem	Ethanolic	Alkaloids (Lophocerine), Flavonoids, Phenols	Mouse lymphoma L5178Y	50-100 mg/kg body weight/day intratumoral route, and 100 mg/kg body weight/day intramuscularly. Survival rate increased significantly and reduced (56.37%) the tumor growth rate	ED50 = 7.8 µg/ml with 63% of cytotoxicity. Lymphoproliferation indice did not differ among the groups treated	Orozco-Barocio et al. (2013)
*Lophophora williamsii*		Methanolic		Wistar rats inoculated subcutaneously into the left flank with C6 cells	1.92 mg/ml of Lophophora williamsii. Reduce tumor growth rate by 42.8%	N.D.	Franco-Molina, et al. (2021)
*Melocactus bellavistensis*	Stem	Methanolic	Alkaloids	Rats with colon cancer induced with dimethylhydrazine (DMH)	1, 5 and 10 mg/kg body weight/day/orally	At concentrations of 10, 5, and 1 mg/kg no neoplasia was observed, and the percentage of dysplasia was 33.3%, 22.2%, and 33.3%, respectively	Ríos-León, et al. (2017)
*Opuntia ficus-indica*	Fruit (Prickly pear)	Aqueous		nu/nu BALB/c mice with SK-OV-3 cells	0.4 ml/day/intraperitoneally. Reduce tumor growth rate by 42.8%, and had no significant effect on body weight loss	Increased annexin IV and decreased VEGF expression in animal tumor tissues	Zou et al. (2005)
	Fruit (Prickly pear)	Methanolic	Indicaxanthin	Mouse melanoma (B16-F10)	3.2 mg/kg orally three/day for 14 days. Reduce tumor growth rate by 86%	Reduction of CXCL1 levels by 42%	Allegra et al. (2018)
	Stem (Cladodes)	Folin polyphenolic method	Phenols and Kaempferol	PC-3 - MCF-7	5 mg/kg body weight/day intraperitoneally. Reduce tumor growth rate by 37.6%, also myeloperoxidase activity and total cholesterol level	Overexpression of cleaved Caspase-9, Hdac11, and Bai1 proteins	Antunes-Ricardo et al. (2021)
	Flour	Alkaline hydrolysis by NaOH	Glycoside isorhamnetin-3-O-glucosylrhamnoside	Tumor growth of HT29 RFP cells xenograft in immunosuppressed mice. Subcutaneous injection	Standard pellet form feed with either 1% (w/w) or 3% (w/w) O.humifusa. Vertical epidermal thickness of the skin were significantly reduced (63.3% and 53.3%)	Increassed antioxidative capacity, superóxido dismutasa (SOD) actiivity and apoptosis. Decreased lipid peroxidation activity	Lee et al. (2012)
*Opuntia humifusa*	Fruit (Prickly pear)	Powder (1 - 3% w/w)		Carcinogenesis induced by 7, 12-dimethylbenz[a] anthracene (DMBA)	Standard pellet form feed with 3% (w/w) O.humifusa. Vertical epidermal thickness of the skin were significantly reduced (50%) and MPO level	COX-2 and iNOS expression were significantly reduced. TNF-a, IL-1b and IL-6 were significantly inhibited. PCNA and cyclin D1were significantly reduced. The level of NF-jB/p65 of the nuclear fraction was significantly decreased	Lee et al. (2013)
	Fruit (Prickly pear)	Ethyl acetate	Flavonoids	Tumor growth of HeLa cells xenograft in immunosuppressed mice. Subcutaneous injection	500 mg/kg body weight/day. Reduced (57.4%) the tumor growth rate, Tissue weights of the lungs, liver, kidneys, spleen, and uterus did not differ among the groups treated	Overexpression of p53 and p21 proteins; reduced the expression of Rb, Cdk4 and cyclinD1 proteins; and levels of Cdk2 and cyclin E did not change	Hahm et al. (2015)
*Pachycereus marginatus*	Stem	Hexane, chloroform, methanol, and methanol-aqueous partition	Lophenol, β-sitosterol, and palmitic acid	Mouse lymphoma L5178Y-R	0.5 mg/kg metOH-aqueous orally. caused 60% survival at 60 days without altering liver parenchyma and enzymes	N.D.	Gomez-Flores et al. (2019)

### 2.6 Genus *Lophophora*

*Lophophora*, a genus of North American cacti, derives its name from the Greek words “lophos” (crest) and “phoros” (bearing), referring to the distinctive brushes of hair on their areoles (Figure 5D). These ornamental plants, which mainly consist of two species, *Lophophora williamsii* (Figure 5C) and *Lophophora diffusa*, grow close to the ground, often in clusters, with spherical, flattened stems known as “buttons” that are 2–7 cm high and 4–12 cm in diameter. Both species flower from March to September and reproduce clonally by forming new shoots from the lower areoles (Bravo Hollis and Sánchez-Mejorada, 1991). *Lophophora williamsii*, commonly known as peyote, has no spines, a blue-green coloration and reaches a diameter of 2–5 inches. The plant is native to central Mexico and is of great cultural and medicinal importance. It is traditionally used to treat various ailments such as infections, arthritis, asthma, influenza, intestinal disorders, diabetes, and snake and scorpion bite (Franco-Molina, et al., 2003). Peyote extracts are known for their effect on the central nervous system and regulate functions such as blood pressure, sleep, hunger and thirst (Shetty, et al., 2012).

Franco-Molina and colleagues (2003) conducted a study demonstrating that the viability of various tumor cell lines, in particular the murine lymphoma cell line L5178Y-R, U937, L929 and MCF7, was significantly reduced by the methanolic extract of *L. williamsii*. In particular, the MCF7 cell line exhibited the highest sensitivity to the cytotoxic effects of the peyote extract. At an extract concentration of 18 μg/mL, the viability of MCF7 was reduced by 1.3%. Simultaneously, the viability of L5178Y-R, U937, and L929 cell lines was reduced by 8%, 45%, and 60%, respectively (Franco-Molina et al., 2003) (Supplementary Table S1). Afterwards, Zhi-Yu and colleagues (2011) investigated the cytotoxic effects of methanolic, hydrochloric, and sodium hydroxide extracts of *L. williamsii* on HeLa cells (a human cervical carcinoma cell line). The *in vitro* MTT analysis revealed that the methanolic and HCl extracts strongly inhibited HeLa cell proliferation in a dose-dependent manner. Interestingly, the NaOH extract had only minimal effects on the HeLa cells. At concentrations of 100 and 150 μg/mL, the extracts induced cell death in over 90% of the cells. The effective concentrations 50 (EC50) for the methanolic and HCl extracts were 4.2 and 7.8 μg/mL, respectively (Zhi-Yu et al., 2011).

Recently, Franco-Molina´s group applied the methanolic extract of Lophophora williamsii (LW) together with the chemotherapeutic agents temozolomide (TMZ) and panobinostat (PAN) to the C6 glia cell line of the mouse brain to investigate the cytotoxic potential of each agent individually and in combination, and to investigate their role in the release of damage-associated molecular patterns (DAMP) by assessing the proteins HMGB1, HSP70 and HSP90, which are characteristic indicators of immunogenic cell death inducers (ICD). All treatments induced cell death: PAN extract and LW induced apoptosis, while TMZ induced both apoptosis and necrosis. The concentrations of HMGB1, HSP70 and HSP90 in the cell lysates did not differ significantly, except for a difference between the LW-treated and non-treated cells. Furthermore, in a rat glioma model, they observed that all treatments decreased tumor volume, but the mechanism of cell death *in vivo* was not ICD. Finally, they indicate that the combination of TMZ, PAN and LW has a cytotoxic effect on glioma cells but does not induce ICD (Franco-Molina, et al., 2021) (Table 1).

### 2.7 Genus *Mammilaria*

**FIGURE 6:**
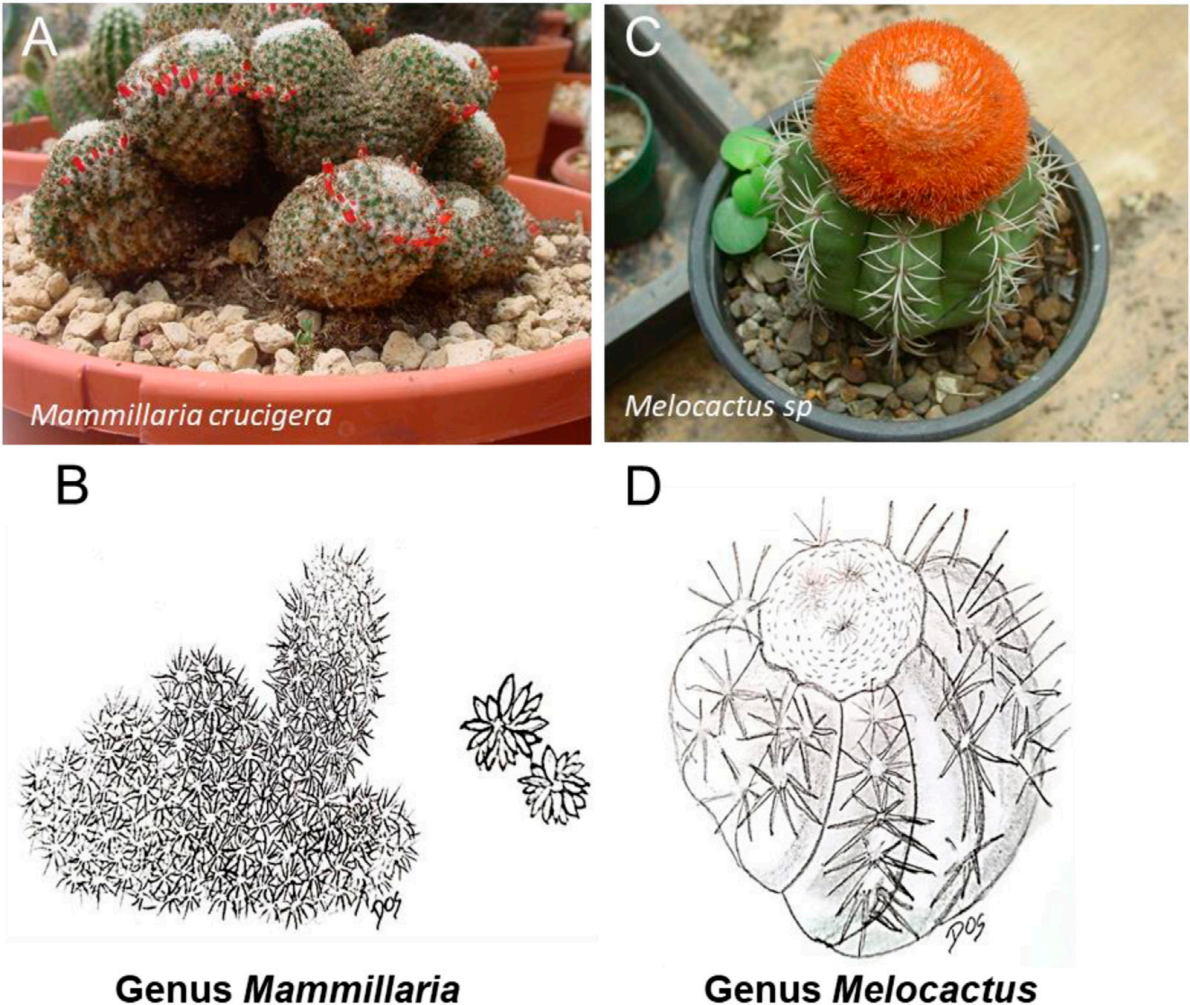
Schematic representative draw **(B, D)** and detailed picture **(A, C)** for the genus *Mammillaria* **(A, B)** and *Melocactus* **(C, D)** of cactaceae family. Mammillaria is the most popular cactus in cultivation. The most common representative of the Cactaceae from the genus *Mammillaria* is *Mammillaria crucigera* commonly called “Owl Eye Cactus”. From the genus *Melocactus* is *Melocactus* zehntneri, also known as the Turk’s cap cactus, or Pope’s head cactus.

*Mammillaria*, a prominent genus within the cactus family (Cactaceae), comprises around 200 species and is characterized by a remarkable morphological diversity (Figure 6B). These species are mainly native to North and Central America, with a particular stronghold in Mexico. Notable representatives include *M. roseoalba, M. magnimamma, M. gaumeri, M. nivosa, M. prolifera,* and *M. uncinata. Mammillaria* cacti typically exhibit globular or occasionally columnar growth forms. Their surface is decorated with a system of tubercles from which a crown of small pink or white flowers emerges (Bravo Hollis and Sánchez-Mejorada, 1991). The juicy, often bright orange-red or purple berries protrude above the tubercles. These berries are 11–17 mm long and 4.5–5.6 mm wide and have a flavor reminiscent of strawberries. Some *Mammillaria* fruits, affectionately known as “chilitos”, owe their name to their dainty shape, reminiscent of chili peppers. Fruit production occurs during two distinct seasons: from January to May and from August to September (Aparicio-Fernandez, et al., 2013).

While *Mammillaria* is a favored choice for ornamental gardens within the Cactaceae family, scientific studies on the bioactivity of its stem extracts remain relatively scarce. In particular, Elansary et al. (2019) delved into the polyphenol profiles of seven *Mammillaria* cacti. Employing a HPLC-DAD method, they qualitatively and quantitatively assessed chosen flavonoids and phenolic acids. Their study also included the evaluation of anticancer, antioxidant and antibacterial properties. The methanolic extracts from the strains of different *Mammillaria* species exhibited antiproliferative activity against several cancer cell lines, including MCF-7, HeLa, Jurkat and HT-29, as well as HEK-293 (normal human cells). The highest activity was observed against HeLa, MCF-7, and Jurkat cells. Among these, the treatments involving *M. rhodantha, M. spinosissima* and *M. muehlenpfordtii* demonstrated the strongest antiproliferative effects. Additionally, the extracts of *M. rhodantha* and *M. spinosissima* exhibited the only anti-cancer activity against HT-29, while the treatments with *M. rhodantha* and *M. spinosissima* were most effective against Jurkat cells (Elansary, et al., 2019) (Supplementary Table S1).

### 2.8 Genus *Melocactus*

*Melocactus*, a genus of cacti, grow either singly or in groups with short cylindrical, occasionally spherical or flattened stems. Typically, they have 9 to 20 straight ribs, rarely spiral. The flowers arise from a well-developed, large, cylindrical, apical cephalium that is narrower than the stem itself. This cephalium bears areoles and sessile spines. The flowers are nocturnal and have simplified structures and colors ranging from pink to other shades. They have a long floral tube, the lower part of which is located between the cephalium elements (Figure 6D). *Melocactus* species are distributed in the Antilles and the continent from Brazil to Mexico. They are commonly known as “Turk’s head” and in Mexico they are also called “viznaga de dulce”. These cacti are used as ornamental plants and, in some regions, they are used to prepare a delicious regional dessert (Bravo Hollis and Sánchez-Mejorada, 1991). The genus *Melocactus* is characterized by a complex mixture of essential oils, flavonoids, steroids, terpenoids, alkaloids, carbohydrates and amino acids (Aquino-Martins et al., 2019; da Silveira Agostini-Costa, 2020) Figure 6A. In Brazil, *Melocactus zehntneri* has long been used in traditional medicine. It has been employed to address respiratory conditions, influenza, physical fragility, throat inflammation, and whooping cough. Additionally, it is associated with “uterine cleaning” (Aquino-Martins et al., 2019; Brandão, et al., 2017; da Silveira Agostini-Costa, 2020).

Research on *Melocactus* has revealed intriguing properties. For instance, supercritical fluid extraction from young *M. zehntneri* plants yielded an alkaloid (or phenethylamine) fraction with activity against *Trichomonas vaginalis* and human vaginal malignant melanoma (HMVII) (Brandão, et al., 2017) (Supplementary Table S1). Various extracts from the pulp of *M. zehntneri* were tested for their effects on cell viability in the murine fibroblast cell line NIH/3T3. While hexane and final water extracts had no significant effect, ethanol, methanol and water extracts exhibited slight effects. Notably, the chloroform extract showed cytotoxicity and reduced cell viability to 68% (Aquino-Martins et al., 2019). *In vivo* evaluation of the alkaloid extract from *Melocactus bellavistensis* in Holtzmann rats with dimethylhydrazine (DMH)-induced revealed no neoplasms at doses of 1.5 and 10 mg/kg. These findings highlight the multifaceted nature of *Melocactus* and its potential applications in both traditional medicine and scientific research (Ríos-León, et al., 2017) (Table 1).

### 2.9 Genus *Myrtillocactus*

**FIGURE 7:**
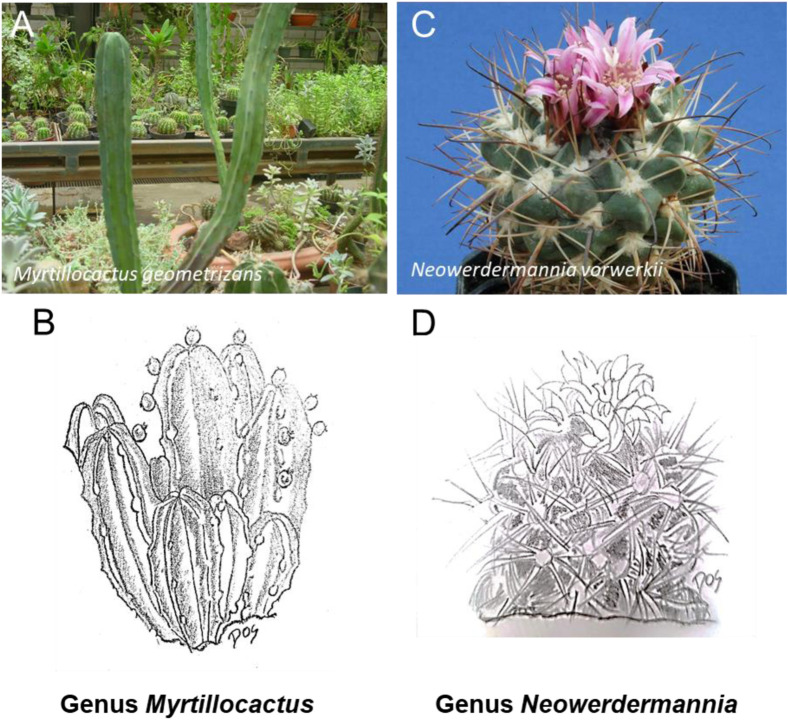
Schematic representative draw **(B, D)** and detailed picture **(A, C)** for the genus *Myrtillocactus* **(A, B)** and *Neowerdermannia* **(C, D)** of cactaceae family. The most common representative of the Cactaceae from the genus *Myrtillocactus* is *Myrtillocactus geometrizans* commonly called blue candle or blue myrtle-cactus. From the genus *Neowerdermannia* is *Neowerdermannia varwerkii*, also known as achakana (Aymara and Quechua).

Native to central and northern Mexico, *Myrtillocactus* sp. is a stately, shrub-like cactus with impressive growth potential. It can reach a height of 4–5 m and has candelabra-like branches Figure 7B. The stems have a diameter of 6–12 cm and have five or six ribs. The flowers, which open in the morning, are white, pale yellow or cream-colored and are pollinated by bees and flies. This fast-growing cactus is resistant to adverse conditions and can tolerate poor soil, light frost and temperatures of up to 45°C. However, it does not thrive in salty soils or in heavy shade. The fruits of this species resemble a berry with a diameter of 1–2 cm. These fruits are commonly known as “garambullo” and are used in various culinary forms: eaten fresh, made into ice cream, popsicles, jam, jelly and even dried as raisins (Figure 7C). They are also mixed with “aguardiente” (a distilled alcoholic drink) to make spirits (Bravo-Hollis and Sánchez-Mejorada, 1978).

Salazar et al. (2011) investigated the sterols isolated from *M. geometrizans* (Figure 7A), in particular peniocerol and macdougallin. These metabolites showed cytotoxicity against several human cancer cell lines, including PC-3 prostate carcinoma, K-562 leukemia, U-251 central nervous system glioma, MCF-7 breast carcinoma, and HCT-15 colon carcinoma. Both peniocerol and macdougallin showed moderate cytotoxic effects with IC50 values between 7.55 and 24.73 µM. Remarkably, peniocerol showed stronger activity than macdougallin in most cancer lines tested, with the exception of K-562 cells where macdougallin showed higher potency (Salazar, et al., 2011) (Supplementary Table S1). Dalla Via et al. (2014) also investigated the cytotoxic effect of peniocerol isolated from the root of *M. geometrizans* on three human tumor cell lines: HeLa (human cervical adenocarcinoma cells), HepG2 (hepatocellular carcinoma) and A-431 (epidermoid carcinoma). Peniocerol showed antiproliferative activity in all cell lines, with IC50 values in the micromolar range. In particular, it showed greater efficacy against HepG2 cells (26.0 µM) compared to HeLa cells (40.1 µM) and A-431 cells (34.6 µM) (Dalla Via et al., 2014) (Supplementary Table S1). In another study, Bolaños and colleagues (2015) administered peniocerol and macdougallin to colon and breast cancer cell lines. These bioactive metabolites showed a dose-dependent reduction in proliferation and apoptosis and effectively disrupted the cell cycle *in vitro* (Bolaños-Carrillo, et al., 2015) (Supplementary Table S1). Recently, Melchor Martínez et al. (2021) investigated the antioxidant capacity and bioactivity of clarified juices from different cactus fruits, including *M. geometrizans, Stenocereus* spp. and *Opuntia* spp. The clarified juice from *M. geometrizans* had the highest concentration of phenolic metabolites and induced cell death in liver and colon cancer cell lines and fibroblasts. The clarified juice from the yellow *Opuntia ficus-indica* fruit showed antioxidant activity and acted selectively on a liver cancer cell line without damaging fibroblasts (Melchor Martínez et al., 2021) (Supplementary Table S1).

### 2.10 Genus *Neowerdermannia*

*Neowerdermannia vorwerkii* Fric, is a solitary cactus native to the Andes region. These low-growing plants have a spherical to depressed spherical shape characterized by strong taproots. Approximately 16 indistinct ribs spiral around the cactus, dividing it deeply into tubercles. The areoles, which are often sunken, are located at the bases of the tips of these tubercles. Spines, some curved or even hook-shaped, adorn the cactus. The flowers, which are located at the tips of the tubercles, open during the day and are funnel-shaped. Their color ranges from white to lilac-pink. The spherical fruits open sideways and release the broadly oval seeds (Figures 7C, D). *Neowerdermannia vorwerkii,* known locally as ‘Achacana’, derives its name from the Quechua dialect, where ‘achakahana’ translates as ‘woman of truth’. Traditionally, this cactus is used by communities in Bolivia, Peru, northern Argentina and northern Chile (Anderson, 2001). as a food (stew) and natural remedy (infusion) for various gastrointestinal complaints, kidney and liver diseases (Chuquimia, et al., 2008; Polini and Camaqui, 2018). In a recent study by Apaza Ticona et al. (2022), an aqueous extract of *N. vorwerkii* yielded three 1,2,4-oxadiazole-type metabolites derived from the n-hexane (HEX) sub-extract. These metabolites were subsequently fractionated with HEX and AcOEt mixtures. Spectroscopic and spectrometric analyzes identified three main metabolites that exhibited cytotoxicity in two tumor cell lines: SK-HEP-1 and Caco-2. Of note, one of them: 3-(pyridin-3-yl)-5-(tiophen-3-yl)-1,2,4-oxadiazole, showed a particularly high cytotoxic effect with CC50 values of 1.48 ± 0.40 μM (SK-HEP-1 cell line) and 1.35 ± 0.54 μM (Caco-2 cell line) (Apaza Ticona, et al., 2022) (Supplementary Table S1).

### 2.11 Genus *Opuntia*

**FIGURE 8:**
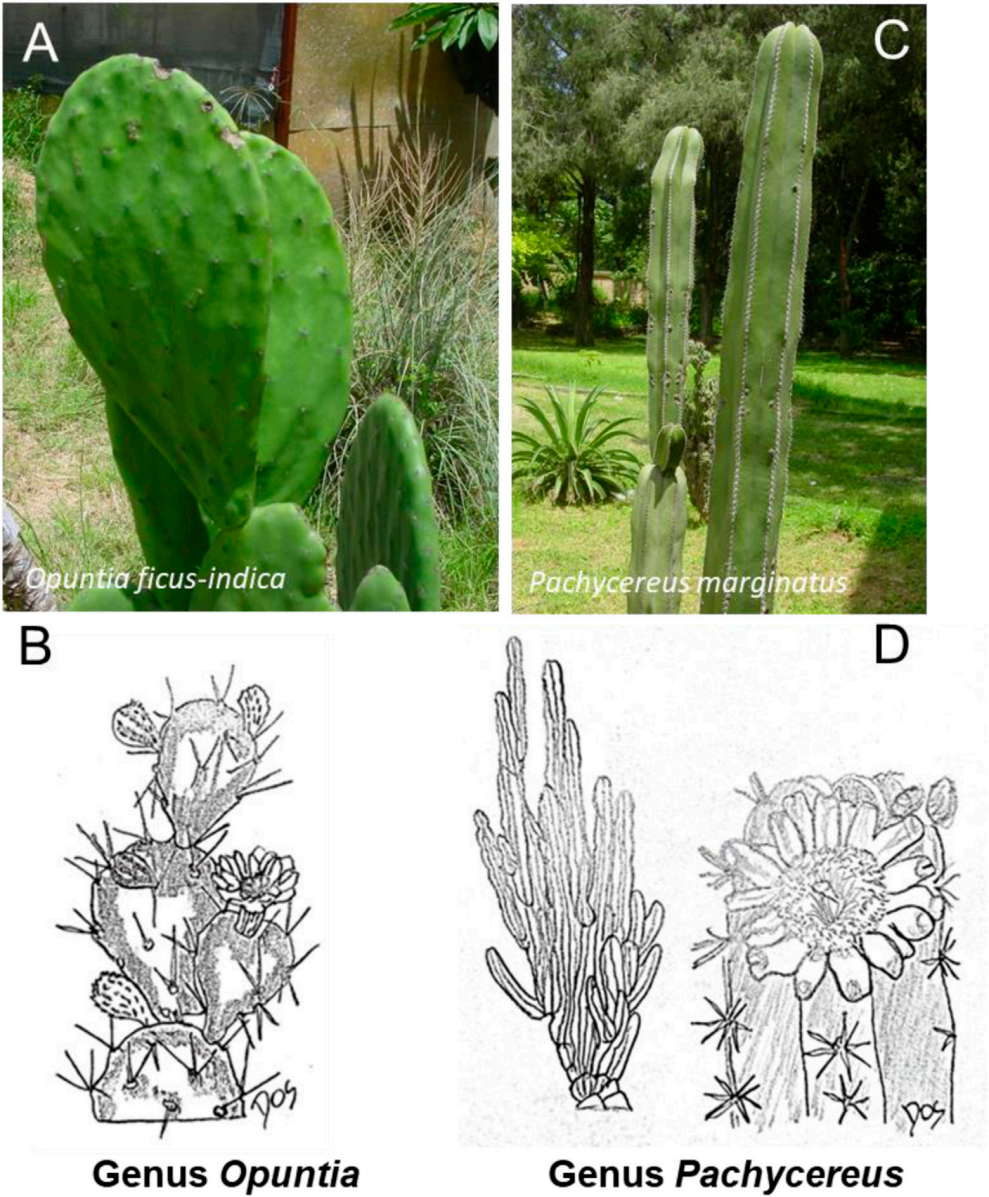
Schematic representative draw **(B, D)** and detailed picture **(A, C)** for the genus Opuntia **(A, B)** and Pachycereus **(C, D)** of cactaceae family. One of the most common and widely recognized representatives of the Cactaceae from the genus *Opuntia* is *Opuntia ficus-indica*, also known as the Prickly Pear Cactus. From the genus *Pachycereus* is *Pachycereus pringlei*, commonly known as the Cardón cactus or Mexican giant cardon.

*Opuntia* spp. commonly known as the prickly pear cactus belongs to the Cactaceae family, is one of the most aboundant and widely distributed genera in America, Africa, Asia, Australia, and in the central Mediterranean area (Bravo-Hollis and Sánchez-Mejorada, 1978; Anderson, 2001; Madrigal-Santillán et al., 2022) (Figure 8B). However, the richest diversity of wild species occurs in Mexico, where at least 126 species have been observed (Bravo-Hollis and Sánchez-Mejorada, 1978; Santos Díaz et al., 2017; Daniloski, et al., 2022; Wang et al., 2023). Having several common names such as “tuna cardona”, “tuna cascarona”, “tuna chaveña”, “tuna mansa”, “tuna tapona and “xoconoxtle (cuaresmeño)” (Ramírez-Rodríguez, et al., 2020; Daniloski, et al., 2022; Madrigal-Santillán et al., 2022). *Opuntia* sp. plants produce edible cladodes, which are oval-shaped and flat shoot systems devoid of leaves. The pulp of their fruits, named as prickly pear, can exhibit various colors depending on ripeness (from white to purple, passing through yellow, green, orange and red). Recently, Jameel et al. (2023) investigated the insecticidal, antimicrobial and anticancer properties of cadmium sulfide nanoparticles (CdS NPs) synthesized from the fruit extract of *Opuntia* sp. They observed a reduction in the viability of pancreatic cancer cells, with viability at a concentration of 200 μg/mL being only 18 and 12 after 24 and 48 h of CdS NP exposure, respectively (Jameel, et al., 2023) (Supplementary Table S1).

*Opuntia ficus-indica* (*O.f-i*) is the most studied species of *cactis*. Their extracts and metabolites showed a plethora of antioxidant, antiviral, antifungal, antibacterial, and anticancer activities. Mainly, their antioxidant activity has been studied as part of their chemopreventive and antigenotoxic potential (Figure 8A). An aqueous acetone extract of Tunisian *O.f-i* syrup showed strong antioxidant potential and also antibacterial activity, being effective against the bacteria *Staphylococcus aureus* and *Staphylococcus epidermidis*, with a minimum bactericidal concentration of 1.3 mg phenols/mL (Dhaouadi, et al., 2013). In addition, acetone, aqueous and ethanol extracts of *Opuntia* stricta cladodes, showed antioxidant activity and anti-inflammatory effects. It is noteworthy that the total phenolic content in all extracts was higher than that of other phytochemicals (Izuegbuna, et al., 2019). The major fatty acid in the oil of *O.f-i* was linolenic acid, followed by oleic acid, linoleic acid, palmitic acid, and stearic acid. The oil also contained total tocopherol (α-, β-, γ-tocopherol) and phytosterols, with campesterol being the major phytosterol, followed by γ- and β-sitosterol and stigmasterol. Being the phenolic metabolites the ones that shows the strongest antioxidant activity (Alqurashi, et al., 2022).

The chemopreventive and antigenotoxic properties of *O.f-i* have been studied in *vitro* models by using different types of extracts, such as: hexane, ethyl acetate (EtOAc), acetone, methanol (MeOH) and aqueous ones, showing promising results (Madrigal-Santillán et al., 2022; Daniloski, et al., 2022). Studies have shown different cytotoxic effects of *O.f-i* extracts on different cancer cell lines. Dhaouadi et al. (2013) showed that an aqueous acetone extract at concentrations ranging from 41.38 to 186.25 μg polyphenols/mL reduced the viability of human SH-SY5Y neuroblastoma and 3T3 fibroblast cells *in vitro* in a dose- and time-dependent manner. While concentrations greater than 106.43 μg/mL resulting in a 50%–60% decrease in cancer cell viability within 1–3 h of treatment. Similarly, Kim et al. (2015) investigated various extracts from *O.f-i* strains for their chemopreventive activities against human SW480 colon and MCF7 breast cancer cells. They found significant cytotoxicity in most extracts, especially in SW480 cells, accompanied by inhibition of cyclooxygenase-2 and increased Bax/Bcl2 ratio (Supplementary Table S1). In addition, Kim et al. observed increased degradation of poly-(ADP-ribose) polymerase, which triggered apoptosis, especially in MCF7 cells treated with *O.f-i* stem extracts. In addition, Abdulazeem et al. (2018) and Das et al., 2021 found cytotoxic activity against MCF-7 and WRL-68 cell lines in alkaloid extracts from dried *Opuntia polyacantha* plants with inhibition rates of 52.7% and 91.89%, respectively, at a concentration of 400 μg/mL. In contrast, Izuegbuna et al. (2019) reported that only the acetone-dried extract showed activity against the U937 cell line at concentrations of 100 and 200 μg/mL. Posteriorly, Alqurashi, et al. showed a differential effect on the cytotoxic activity of the *O.f-i* oil over Vero cells (derived from the kidney of the African green monkey), the PC-3 cell line (prostate carcinoma), and the A2780 cell line (ovarian carcinoma). Revealing that oil exhibited highest cell viability (99.63% ± 0.45%) at 250 μg/mL of oil against Vero cells. In the case of A-2780 cell line, *O.f-i* oil showed weak inhibitory activity. Although in the case of PC-3 cell line, *O.f-i* oil showed significant inhibitory activity (70% and 83% at 250, and 500 μg/mL of oil respectively) (Alqurashi, et al., 2022). At that time, Ali et al. (2022) evaluated the cytotoxicity of different concentrations (1, 10, 100, 200, 300, 400 μg/mL) for cyclohexanone (OFICE), 70% (OFI70EE) and 100% (OFI100EE) ethanol of *O.f-i* peel (OFIPs) extracts, against MCF-7 cell line detected by sulforhodamine B (SRB) assay. They showed a high dose of OFICE, OFI70EE, and OFI100EE highly significantly (*p* < 0.05) reduced cell viability of MCF-7 cancer cells at a concentration of 400 μg/mL after 72 h. Respecting the effect of three *O.f-i* extracts on cells exhibits dose-dependent toxicity and that OFICE at different concentrations showed significantly reduced cell viability than the ethanol extracts and the former have a greater effect as antitumor (Ali, et al., 2022). These results underline the diverse anticancer potential of *Opuntia* extracts in different cell types and extraction methods.

Some works have been demonstrated anti-cancer properties from *O. ficus* extracts. Using an aqueous extract of prickly pear (*O.f-i*) on ovarian, cervical and bladder cancer cells and tumor growth in nu/nu Balb/C mice, Zou et al., have shown that after a 1-, 3-, and 5-day treatment of the cells, a dose-dependent and time-dependent inhibition of cell growth and an induction of apoptosis was observed. In addition, the extracts showed a significant suppression of tumor growth and an upregulation of annexin IV expression in the animals. Furthermore, they affected the cell cycle of cancer cells by promoting G1 phase arrest and decreasing G2 and S phases (Zou et al., 2005) (Supplementary Table S1, Table 1). On another studies, Feugang et al. (2010) showed that an aqueous extract of prickly pear cactus increases oxidative stress by increasing the concentration of reactive oxygen species (ROS) and inducing DNA methylation of p16 and RASSF-1A in bladder cancer cells (Feugang, et al., 2010). Additionally, it is capable of eliciting a significant degree of DNA fragmentation in ovarian cancer cells (OVCA420) (Feugang, M., et al., 2010). In both, changes in the expression of apoptosis-related genes, such as: Bax, Bad, caspase 3, Bcl2, p53 and p21, that respond to ROS were detected. Likewise, a decrease in NF-kappa B expression was observed in conjunction with an increase in p-AKT expression. Also, Serra and coworkers (2013) using an hydroalcoholic extracts of *O.f-i* and *Opuntia robusta* evaluated their antiproliferative effect in a human colon carcinoma HT29 cell line. The results showed that the natural extracts efficiently inhibited cancer cell growth and induced cell cycle arrest in different checkpoints G1, G2/M, and S. The phytochemical metabolites present in the samples, namely, betacyanins, flavonoids and phenolic acids were identified as the main responsible for the cell cycle arrest (Serra, et al., 2013). More recently, Ortíz-González et al. (2022) used the big data platform Cellulat to model the PI3K/Akt/mTOR signaling pathway, which is critical for cell cycle regulation and cancer cell survival. Their aim was to predict the targets affected by *O.joconostle* extract and determine the concentration range for *in vitro* experiments on breast cancer cells. *In silico* analysis revealed that the extract inhibits cell proliferation, regulates the cell cycle and induces apoptosis via the PI3K/Akt/mTOR pathway. *In vitro* experiments showed the antiproliferative effect of the aqueous crude extract by arresting the cell cycle in the G2/M phase (Ortíz-González, et al., 2022).

Different types of *O.f-i* seed oils exhibited apoptotic induction effects on colon adenocarcinoma cell lines. Becer et al. investigated, in a couple of studies, the fatty acid content and apoptotic effect of cactus pear seed oils (CPS) with and without spines of *O.f-i* on colon adenocarcinoma cell lines. They observed activity against Colo-320 and Colo-741 cells with CPS oil with spines, while CPS oil without spines significantly reduced the number of viable cells in both lines. Using the TUNEL assay, they found a significantly higher number of TUNEL-positive cells in Colo-320 cells treated with CPS without spines than in the control group. In another study, they investigated the antiproliferative effect of CPS oils on primary (Colo-320) and metastatic (Colo-741) colon adenocarcinoma cell lines. They found a reduction in the proliferation of Colo-320 cells, together with a protective effect on molecular mechanisms related to angiogenesis. Interestingly, thornless CPS oil increased angiogenesis through signaling molecules, while spiky CPS oil decreased signaling molecules involved in angiogenesis. Both types of CPS oils increased TNF-α expression in Colo-320 cells, but not IL-6, indicating no significant changes in the tumor microenvironment (Becer et al., 2018; 2021). Meanwhile, Heikal et al. (2021) compared the anticancer effects of extracts from *O.f-i* cladodes cultured *in vitro* and *in vivo* on PC3 prostate cancer and Mcf7-7 breast cancer cell lines. They showed that *in vitro* micropropagated cladodes produced more phenols and kaempferol than those cultured *in vivo*, with a dose-dependent increase in cytotoxicity observed in both PC3 and Mcf7 cells (Heikal, et al., 2021) (Supplementary Table S1).

While betacyanins, flavonoids, and phenolic acids have been identified as responsible for the antiproliferative effects of the extracts (Serra et al., 2013; Aruwa et al., 2018), various studies have investigated the antitumor properties of phytochemicals extracted from different parts of *O.f-i*. Sreekanth et al. (2007) investigated the antiproliferative effect of betanin on chronic myeloid leukemia cells and found a reduction in cell proliferation and induction of apoptosis. Li et al. (2014) demonstrated the growth inhibition of lung squamous cell carcinoma cells by polysaccharides extracted from *Opuntia dillenii*, suggesting cell cycle inhibition and induction of apoptosis. Antunes-Ricardo et al. (2014) investigated the cytotoxicity of extracts and glycosides from *O.f-i* on colon cancer cells and emphasized their differential effects on cell viability and induction of apoptosis. In their subsequent studies, they elucidated the mechanisms involved in apoptosis induction by isorhamnetin-3-O-glucosylpentoside (IGP) and demonstrated caspase-dependent mitochondrial damage in colon cancer cells. In addition, they demonstrated the growth inhibitory effect of *O.f-i* extract and its glycoside isorhamnetin-3-O-glucosylrhamnoside (IGR) on colorectal adenocarcinoma cells and their effects on apoptosis induction and tumor growth reduction in a mouse model. Another compound, indicaxanthin, extracted from the fruit of *O.f-i*, showed significant inhibitory effects on human melanoma cells both *in vitro* and *in vivo* by inducing apoptosis and reducing cell invasiveness (Antunes-Ricardo et al., 2019; 2021) (Supplementary Table S1, Table 1). Meanwhile, Lefsih et al. (2018) discovered the cytotoxic activity of pectin extracts on LAN5 cancer cells without affecting normal cells, suggesting their potential as selective anticancer agents. Overall, these results highlight the promising anticancer properties of *O.f-i*-derived metabolites in various cancer types and provide insights into their mechanisms of action (Lefsih, et al., 2018) (Supplementary Table S1).

Summarizing, *O.f-i* and its various extracts and metabolites show promising potential in cancer prevention and treatment through multiple mechanisms, including antioxidant activity, apoptosis induction, and cell cycle arrest.

### 2.12 Genus *Pachycereus*

The genus *Pachycereus*, which belongs to the tribe *Pachycereae* in North America, comprises five species of tree-like cacti that are mainly found in western Mexico, with some extensions to Baja California, Sonora and Chiapas. *Pachycereus* is characterized by its columnar and candelabra-like shape and is known by various names such as cardón, organ or candelabra (Figure 8D). *Pachycereus weberi*, for example, can reach a height of up to 15 m, with trunks 60–200 cm tall and 30–60 cm in diameter, with gray bark and persistent spines. This species is native to southern and central Mexico, especially in the arid and semi-arid regions of Puebla, Guerrero, Morelos and Oaxaca. Its fruit, which is often referred to as “chico fruit” or “tuna de cardón”,“ is usually used to make refreshing fruit drinks by pureeing it and adding water (Arias and Terrazas, 2009).

Various extracts from the fruit juice of *Pachycereus marginatus* (Figure 8C) and *P. weberi* were tested for their cytotoxic effect against different cancer cell lines. Extracts from *P. marginatus* showed significant inhibition against murine lymphoma L5178Y-R and skin melanoma cells B16F10, with methanolic and hexane extracts exhibiting high cytotoxicity (Quintanilla-Licea, et al., 2016). The fruit juice of *P. weberi* showed cytotoxic activity against four different mammalian cancer cell lines: Mammary carcinoma (MCF-7), prostate (PC3), colon (CaCo-2) and liver (HepG2), specifically damaging colon CaCo2 and mammary carcinoma MCF-7 cells (Sandate-Flores, et al., 2020). In addition, studies with *P. marginatus* extract and isolated metabolites such as lophenol, β-sitosterol and palmitic acid showed promising cytotoxic effects against lymphoma cells *in vitro* and improved survival rates in tumor-bearing mice.

In a different work, the cytotoxic effect of different extracts and metabolites from *P. marginatus* on L5178Y-R lymphoma cells was investigated. Hexane, chloroform and methanol extracts showed varying degrees of cytotoxicity, with significant percentages ranging from 20% to 85%, 32%–84% and 32%–72%, respectively. The extract of methane and the aqueous partition also showed significant cytotoxicity, ranging from 65% to 78% (Hernández-Martínez, et al., 2016). Later on, another study investigated the effects of the *P. marginatus* extract and the isolated compounds, including lophenol, β-sitosterol and palmitic acid, on mice with tumors. Oral administration of the aqueous partition extract resulted in a 60% survival rate at 60 days, with no adverse effects on liver function or histopathology. In addition, the n-hexane extract and metabolites showed considerable cytotoxicity against L5178Y-R cells *in vitro*, indicating their potential as anticancer agents (Gomez-Flores, et al., 2019) (Table 1). Recently, Aispuro-Hernández and colleagues evaluated the nutritional quality and antiproliferative capacity of the fruit juice of *Pachycereus pecten-aboriginum* and *P. pringlei* in cervical cancer (HeLa) and breast cancer (MDA-MB-231, MCF-7 and T-47D) cell lines. The results showed that the fruit juices of both cacti are rich in polyphenols, flavonoids, betalains, vitamin C and myo-inositol and have antioxidant and anticancer potential by inhibiting the proliferation of all cell lines studied, with IC50 values ranging from 198 to 287 μg gallic acid equivalents/mL (Aispuro-Hernández, et al., 2023).

### 2.13 Genus *Pereskia*

**FIGURE 9:**
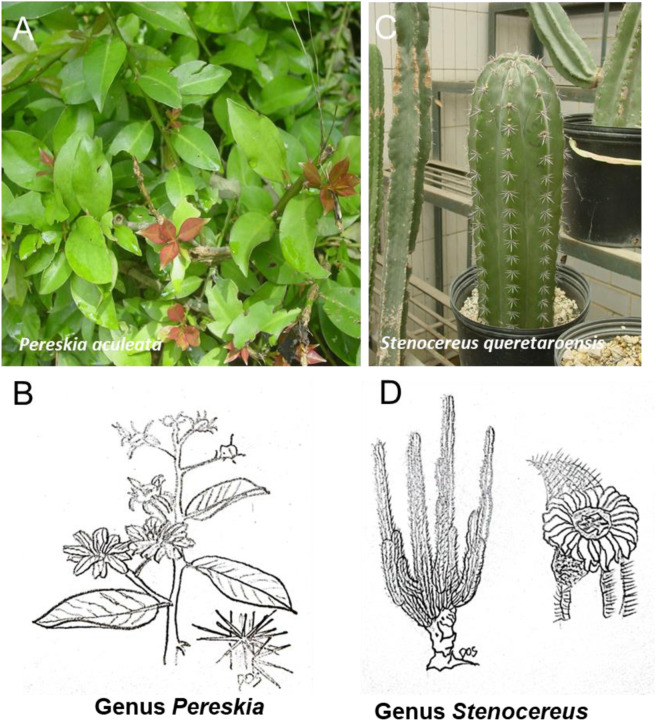
Schematic representative draw **(B, D)** and detailed picture **(A, C)** for the genus Pereskia **(A, B)** and Stenocereus **(C, D)** of cactaceae family. One of the most common representatives of the Cactaceae from the genus *Pereskia* is *Pereskia aculeata*. Also known as Barbados gooseberry or leaf cactus. From the genus *Stenocereus* is *Stenocereus pruinosus*, also known as the Organ Pipe Cactus.

The genus *Pereskia*, which comprises 17 species, is characterized within the cactus family (Cactaceae) by its unique features, which include regular leaf development and green leaves. Native to regions between Brazil and Mexico, *Pereskia* plants have large leaves and thin stems, unlike typical cacti (Figure 9B). Historically, species such as *Pereskia aculeata* (Figure 9A) and *Pereskia grandifolia*, known as ora-pro-nobis, were used to decorate colonial-era churches in Brazil (da Silveira Agostini-Costa, 2020). In addition to its use as an ornamental plant, *Pereskia* also has a long history in traditional medicine. Various species are used to treat diseases such as cancer, high blood pressure, diabetes and stomach problems. In addition, the *pereskia* leaves, which are known for their high protein content, are consumed as leafy vegetables in Brazil. They contain essential amino acids and fulfill the nutritional requirements according to FAO standards. These leaves have various biological activities, including anti-inflammatory, antimicrobial and anti-cancer properties, which make them valuable in both traditional and modern medicine (Sharif, et al., 2013; da Silveira Agostini-Costa, 2020).

Some *Pereskia* species, including *P. grandifolia, P. aculeate, Pereskia sacharosa* and *Pereskia bleo*, have been studied for their cytotoxic effects on various cancer cell lines, with particular attention given to *P. bleo*. Studies by Malek NurestriAS. et al. (2009) revealed that the hexane extract of *P grandifolia* (Haw) exhibited the highest cytotoxicity on the KB cell line, while the ethyl acetate extract showed significant cytotoxic effects on both KB and MCF7 cell lines. In addition, seven isolated metabolites from *P. grandifolia* (Haw) were tested for cytotoxic activity, with 2,4-di-tret-butylphenol showing similar cytotoxicity to *P. bleo* (Kunth). Malek et al. performed similar studies with methanolic and fractionated extracts of *P. grandifolia* (Haw) on a human lung carcinoma cell line (A549). In addition, Pinto et al. (2012) investigated the cytotoxic activity of the crude methanolic extract and different fractions from the leaves of *P. aculeate* (Miller) on human promyelocytic leukemia (HL60) and human breast adenocarcinoma (MCF-7) cell lines and observed inhibition of the cancer cell line without affecting normal cells. In addition, Asmaa et al. (2014) demonstrated the proliferation inhibitory effect of ethanolic extract of *P. sacharosa* (Griseb) on two leukemic cell lines, MV4-11 and K562, by inducing intrinsic apoptotic pathways and cell cycle arrest with upregulation of apoptosis and cell cycle-related regulatory proteins (Asmaa, et al., 2014).

Research on *P. bleo* (Kunth) has demonstrated its potential cytotoxic effect on various cancer cell lines. Tan et al. (2005) found that the methanol extract of *P. bleo* (Kunth) showed a significant cytotoxic effect on human breast cancer cells T-47D, with saponin and complex glycosides being responsible for the high cytotoxicity. This study also illustrated the mechanism of cell apoptosis and concluded that the activation of caspase-3 and c-myc pathways was the route of cell death. Similarly, Er et al. (2007) applied both methanolic and aqueous extracts of *P. bleo* (Kunth) to mouse breast cancer cell line (4T 1) and a normal mouse fibroblast cell line (NIH/3T3), and observed significant anti-proliferative effects under different conditions. The result of this study also contradicted the study by Tan et al. The authors suggested two possible reasons for this contradiction: 1) The metabolites of the plant extract responsible for the cytotoxic activity may not be concentrated in the leaves, as the extracts of the first study were obtained from leaves and stems of the plants, while only leaves were used in the second study. 2) The content of fetal bovine serum was different in both tests (Er, et al., 2007).

Malek et al. (2008) investigated the cytotoxic activity of methanolic and fractionated extracts (hexane, ethyl acetate, and water) of *P. bleo* (Kunth) on different human carcinoma cell lines, with the ethyl acetate and methanol extracts showing significant cytotoxicity against the KB cell line (Malek, et al., 2008). On the other hand, five isolated bioactive metabolites (dihydroactinidiolide, 2,4-di-tret-butylphenol, α-tocopherol, β-sitosterol and phytol) from the ethyl acetate extract of *P. bleo* (Kunth) were individually evaluated against five cancer cell lines. But only 2,4-ditret-butylphenol showed remarkable inhibition and α-tocopherol was well cytotoxic against different cell lines Ethyl acetate and methanol extract of *P. bleo* (Kunth) showed the most significant cytotoxicity against only 1 cell line (KB), while the hexane fraction exerted a moderate cytotoxic effect on the same cell line. The interesting result was that the methanolic crude extract and all fractionated extracts had no damaging effect on the non-cancer cell line (MRC-5) (Malek, et al., 2009a). Conversely, Liew et al. (2012) found moderate cytotoxicity of crude methanol extracts of *P. grandifolia* (Haw) against hypoxic cancer cells through inhibition of HIF (Hypoxia Inhibition Factor) activity, while extracts of *P. bleo* (Kunth) showed no cytotoxic effect.

More recently, Siew et al. (2019) investigated the antiproliferative activity of *P. bleo* (Kunth) extracts in different cancer cell lines and obtained promising results. They used three different solvents (water, ethanol, and methanol) from seven medicinal plants, including *P. bleo*, over twelve human cancer cell lines. In addition, Mohd-Salleh et al. (2020a) in a couple of studies identify the phytochemicals present in the crude extracts of *P. bleo* (Kunth) leaves by using hexane, ethyl acetate, methanol, and aqueous via Gas chromatography mass spectrometry (GCMS) technique.

Mohd-Salleh et al. (2020b) conducted a study to identify phytochemicals present in crude extracts of *P. bleo* (Kunth) leaves and assesed their cytotoxic effect on selected cancer cell lines. The ethyl acetate extract demonstrates significant cytotoxicity against cervical and breast cancer cells. Furthermore, this extract induced cell cycle arrest and apoptosis in cervical cancer cells (Siew, et al., 2019). Specifically, the ethyl acetate extract of *P. bleo* leaves exhibited potent cytotoxic effect on HeLa cells at IC50 value of 17.51 ± 8.6 μg/mL, and showed activity against MDA-MB-231 cells at 19.39 ± 1.26 μg/mL. Meanwhile, it exhibited a moderate cytotoxic effect on SW480 cells with an IC50 value of 31.80 ± 16.1 μg/mL (Mohd-Salleh, et al., 2020a). In a separate study, the same researchers investigated the effects of the ethyl acetate extract of *P. bleo* leaves (PBEA) on inducing cell death through cell cycle arrest and apoptosis in cervical cancer cells (HeLa), using flow cytometry. Their findings reveal that PBEA caused cell cycle arrest at the G0/G1 phase and upregulated apoptotic protein levels of Bax and p53 while downregulating Bcl-2 in a time-dependent manner over a 72-h treatment period (Mohd-Salleh, et al., 2020b). Overall, these findings underscore the potential of *P. bleo* (Kunth) as a promising source of cytotoxic metabolites for cancer therapy.

### 2.14 Genus *Stenocereus*

*Stenocereus* (A. Berger) Riccob, commonly referred to as organ pipe cactus, is a columnar cactus species with a wide geographic distribution spanning from the southwest United States of America to Venezuela in South America Figures 9B–D. It comprises 24 species, of which around 80% are found in various Mexican states such as Jalisco, Nayarit, Colima and Oaxaca (Bravo-Hollis and Sánchez-Mejorada, 1978; Ramírez-Rodríguez, et al., 2020; García-Cruz, et al., 2022). The plant has columnar branches that grow from the ground. The stems grow 15–20 cm thick and up to 5 m high and often have no branches, hence its nickname. It produces pink flowers and edible fruit known as “piraya”. They are characterized by deciduous areoles that contain pulp in shades of red, yellow, orange, purple, white or pink, along with small black seeds (Bravo-Hollis and Sánchez-Mejorada, 1978; Harlev, et al., 2013; Ramírez-Rodríguez, et al., 2020). These fruits have a low acidity, a high soluble solids content and a sweet taste. Typically, the fruit is spherical to ovoid, 5–10 cm long and is harvested twice a year, from April to May and from September to October (Pimienta-Barrios and Nobel, 1994; Ramírez-Rodríguez, et al., 2020). Ethnobotanical studies indicate various traditional uses of piraya, including use against rheumatism, insect bites, snake bites, hemorrhages, gastrointestinal problems, diabetes mellitus, hypertension, and to protect the immune system (Ramírez-Rodríguez, et al., 2020). In particular*, S. thurberi,* a species from the genus *Stenocereus*, is known for its effectiveness in treating skin cancer and skin lesions and is often used for this purpose in traditional Mexican medicine (Harlev, et al., 2013).

Melchor Martínez et al. (2021) investigated the cytotoxic effect of five clarified juices extracted from different cactus fruits on four different mammalian cancer cell lines, including MCF-7, PC3, Caco-2 and HepG2, as well as on a normal cell line NIH/3T3. They observed a significant decrease of 70% in the viability of HepG2 and PC3 cells when treated with the juices from red and yellow pitayas of *S. spp*. respectively. However, these juices showed no antiproliferative effect on MCF-7 and Caco-2 cells. Conversely, the proliferation of normal NIH/3T3 cells was inhibited by 43% and 55% when exposed to the juice of yellow and red pitayas, respectively (Melchor Martínez et al., 2021) (Supplementary Table S1).

## 3 Conclusion and perspectives

The surge in life expectancy has led to an increased prevalence of chronic degenerative diseases, particularly cancer. This trend represents a significant challenge to public health, given the high mortality rates associated with these conditions. Chemotherapy, a critical component of cancer treatment, is hampered by the phenomenon of multidrug resistance, highlighting the need for alternative therapeutic strategies. Natural products, particularly those derived from plant sources such as cacti, are emerging as potential solutions to these challenges. Their historical medicinal use and burgeoning scientific research provide compelling evidence of their potential. The cacti family, a diverse group of succulent plants, harbors a wealth of bioactive metabolites that exhibit potential anti-cancer properties. However, research into the chemopreventive and anticancer capabilities of cacti is still in its early stages. Although there are more than 1,500 species, very few have been studied. In this review, we report on 14 genera and 36 species with which anticancer studies have been conducted. On the other hand, most of these studies have been conducted with crude extracts of fruits, leaves and stems, and very few pure metabolites have been determined, and even less studies have investigated the molecular or immunological mechanisms of action (Supplementary Table S1). This review, based on an extensive literature search, is intended to shed light on experimental efforts investigating the role of cacti as potential anticancer agents. It is important to point out that most of these studies have been conducted *in vitro* with tumor lines of human origin, where experimental conditions are tightly controlled, and many systemic physiological variables are not checked or measured. For example, it is not known whether the concentrations used *in vitro* can be reproduced *in vivo* or whether there are side effects in other systems, and only a very limited number have been extended to animal models. As we look towards the future, further research into the therapeutic potential of cacti and other natural products holds promise for the development of novel anti-cancer agents. These agents may have improved efficacy and reduced side effects compared to current treatments, which would represent a significant advance in the field of cancer therapeutics.

## References

[B1] Abdulazeem L. Al-Alaq F. T. Alrubaei H. A. Al-Mawlah Y. H. Alwan W. K. (2018). Anti-cancer activity of Opuntia polyacantha alkaloid extract on human breast cancer cell line. J. Pharm. Sci. Res. 10 (7), 1753–1754.

[B4] Aispuro-Hernández E. de Jesús Vergara-Jiménez M. Cárdenas-Torres F. I. Lagarda-Díaz I. Martínez-Téllez M. Á. Soto-Córdova F. J. (2023). Fruit juices of etcho (Pachycereus pecten-aboriginum) and giant cardon (Pachycereus pringlei) are sources of health-promoting ingredients with potential anticancer properties. Plant foods Hum. Nutr. Dordrecht, Neth. 78 (4), 728–734. 10.1007/s11130-023-01099-x 37658958

[B5] Ali S. K. Mahmoud S. M. El-Masry S. S. Alkhalifah D. H. M. Hozzein W. N. Aboel-Ainin M. A. (2022). Phytochemical screening and characterization of the antioxidant, antiproliferative and antibacterial effects of different extracts of Opuntia ficus-indica peel. J. King Saud Univ. – Sci. 34, 102216. 10.1016/j.jksus.2022.102216

[B6] Allegra M. De Cicco P. Ercolano G. Attanzio A. Busà R. Cirino G. (2018). Indicaxanthin from Opuntia ficus-indica (L. Mill) impairs melanoma cell proliferation, invasiveness, and tumor progression. Phytomedicine Int. J. phytotherapy Phytopharm. 50, 19–24. 10.1016/j.phymed.2018.09.171 30466978

[B7] Allegra M. Tutone M. Tesoriere L. Almerico A. M. Culletta G. Livrea M. A. (2019). Indicaxanthin, a multi-target natural compound from Opuntia ficus-indica fruit: from its poly-pharmacological effects to biochemical mechanisms and molecular modelling studies. Eur. J. Med. Chem. 179, 753–764. 10.1016/j.ejmech.2019.07.006 31284085

[B8] Alqurashi A. S. Al Masoudi L. M. Hamdi H. Abu Zaid A. (2022). Chemical composition and antioxidant, antiviral, antifungal, antibacterial and anticancer potentials of Opuntia ficus-indica seed oil. Molecules 27 (17), 5453. 10.3390/molecules27175453 36080220 PMC9457745

[B9] Anderson E. F. (2001). The cactus family, pentland, Oregon. Timber Press, 483. ISBN 978-0-88192-498-5.

[B10] Antunes-Ricardo M. Guardado-Félix D. Rocha-Pizaña M. R. Garza-Martínez J. Acevedo-Pacheco L. Gutiérrez-Uribe J. A. (2021). Opuntia ficus-indica extract and isorhamnetin-3-O-glucosyl-rhamnoside diminish tumor growth of colon cancer cells xenografted in Immune-suppressed mice through the activation of apoptosis intrinsic pathway. Plant foods Hum. Nutr. 76, 434–441. 10.1007/s11130-021-00934-3 34786663

[B11] Antunes-Ricardo M. Hernández-Reyes A. Uscanga-Palomeque A. C. Rodríguez-Padilla C. Martínez-Torres A. C. Gutiérrez-Uribe J. A. (2019). Isorhamnetin glycoside isolated from Opuntia ficus-indica (L.) MilI induces apoptosis in human colon cancer cells through mitochondrial damage. Chemico-biological Interact. 310, 108734. 10.1016/j.cbi.2019.108734 31276661

[B12] Antunes-Ricardo M. Moreno-García B. E. Gutiérrez-Uribe J. A. Aráiz-Hernández D. Alvarez M. M. Serna-Saldivar S. O. (2014). Induction of apoptosis in colon cancer cells treated with isorhamnetin glycosides from Opuntia ficus-indica pads. Plant foods Hum. Nutr. Dordrecht, Neth. 69 (4), 331–336. 10.1007/s11130-014-0438-5 25186940

[B13] Aparicio-Fernandez X. Loza-Cornejo S. Torres-Bernal M. G. Velazquez-Placencia N. D. (2013). Chemical and morphological characterization of Mammillaria uncinate (Cactaceae) fruits. J. Prof. Assoc. Cactus Dev. 15. 32–41 < Go to ISI > ://WOS:000341274600004.

[B14] Apaza Ticona L. Rumbero Sánchez Á. Humanes Bastante M. Serban A. M. Hernáiz M. J. (2022). Antitumoral activity of 1,2,4-oxadiazoles compounds isolated from the Neowerdermannia vorwerkii in liver and colon human cancer cells. Phytochemistry 201, 113259. 10.1016/j.phytochem.2022.113259 35662550

[B123] Aquino-Martins V. G. Q. Melo L. F. M. Silva L. M. P. Targino de Lima T. R. Fernandes Queiroz M. Viana R. L. (2019). In Vitro Antioxidant, Anti-Biofilm, and Solar Protection Activities of Melocactus zehntneri (Britton and Rose) Pulp Extract. Basel, Switzerland: Antioxidants 8 (10), 439. 10.3390/antiox8100439 PMC6826963 31581486

[B15] Argueta A. Cano Asseleih L. M. Rodarte Garcísa M. E. (1994). “Atlas de Las Plantas de La Medicina Tradicional Mexicana,” in Biblioteca de la medicina tradicional mexicana. Editors Argueta A. Gallardo Vázquez M. C. (México: D.F: Instituto Nacional Indigenista). 978-968-29-7323-9.

[B16] Arias S. Terrazas T. (2009). Taxonomic revision of Pachycereus (cactaceae). Syst. Bot. 34, 68–83. 10.1600/036364409787602384

[B17] Aruwa C. E. Amoo S. O. Kudanga T. (2018). Opuntia (Cactaceae) plant compounds, biological activities and prospects – a comprehensive review. Food Res. Int. 112, 328–344. 10.1016/j.foodres.2018.06.047 30131144

[B18] Ashaduzzaman Nur Md. Rasel Uddin M. Sultana Meghla N. Jashim Uddin M. Ziaul Amin M. (2023). *In vitro* anti-oxidant, anti-inflammatory, anti-bacterial, and cytotoxic effects of extracted colorants from two species of dragon fruit (Hylocereus spp.). Food Chem. Adv. 2, 100318. 10.1016/j.focha.2023.100318

[B19] Asmaa M. J. Al-Jamal H. A. Ang C. Y. Asan J. M. Seeni A. Johan M. F. (2014). Apoptosis induction in MV4-11 and K562 human leukemic cells by Pereskia sacharosa (Cactaceae) leaf crude extract. Asian Pac. J. cancer Prev. APJCP 15 (1), 475–481. 10.7314/apjcp.2014.15.1.475 24528077

[B20] Becer E. Kabadayi H. Mericli A. H. Kivancli B. Vatansever H. S. Mericli F. (2021). Fatty acid composition of Opuntia ficus-indica seed oil control angiogenic activity in colon carcinoma cell lines (Article). Prog. Nutr. 23 (2), 12. Article e2021051. 10.23751/pn.v23i2.10042

[B21] Becer E. Kabadayı H. Meriçli F. Meriçli A. H. Kıvançlı B. Vatansever S. (2018). “Apoptotic effects of Opuntia ficus indica L. seed oils on colon adenocarcinoma cell lines,” in Multidisciplinary digital publishing Institute (MDPI) proceedings, 2, 1566. 10.3390/proceedings2251566

[B22] Bolaños-Carrillo M. A. Ventura-Gallegos J. L. Saldivar-Jiménez A. D. Zentella-Dehesa A. Martínez-Vázquez M. (2015). Effect of sterols isolated from Myrtillocactus geometrizans on growth inhibition of colon and breast cancer cells. Evidence-based complementary Altern. Med. eCAM 2015, 589350. 10.1155/2015/589350 PMC4465765 26113867

[B124] Brandão G. H. A. Rigo G. Roque A. A. Souza A. C. D. Scopel M. Nascimento C. A. O. (2017). Extraction of bioactive alkaloids from Melocactus zehntneri using supercritical fluid. J. Supercritical Fluids. 129, 28–35. 10.1016/j.supflu.2016.12.012

[B23] Bravo-Hollis H. Sánchez-Mejorada H. R. (1978). Las cactáceas de México Vol 1. Universidad Nacional Autónoma de México UNAM.

[B24] Bravo Hollis H. Sánchez-Mejorada H. R. (1991). Las cactáceas de México. Tercera ed. Vol. 2. Universidad Nacional Autónoma de México. (UNAM).

[B25] Bray F. Laversanne M. Sung H. Ferlay J. Siegel R. L. Soerjomataram I. (2024). Global cancer statistics 2022: GLOBOCAN estimates of incidence and mortality worldwide for 36 cancers in 185 countries. CA a cancer J. Clin. 74 (3), 229–263. 10.3322/caac.21834 38572751

[B26] Bukowski K. Kciuk M. Kontek R. (2020). Mechanisms of multidrug resistance in cancer chemotherapy. Int. J. Mol. Sci. 21 (9), 3233. 10.3390/ijms21093233 32370233 PMC7247559

[B28] Choudhari A. S. Mandave P. C. Deshpande M. Ranjekar P. Prakash O. (2020). Phytochemicals in cancer treatment: from preclinical studies to clinical practice. Front. Pharmacol. 10, 1614. 10.3389/fphar.2019.01614 32116665 PMC7025531

[B29] Chuquimia F. Alvarado J. A. Penarrieta J. M. Bergenståhl B. Åkesson B. (2008). Determinación de la capacidad antioxidante y la cuantificación de compuestos fenolicos y flavonoidicos de cuatro especies vegetales de la region Andina de Bolivia. Rev. Bol. Quim. 25, 76–84.

[B30] Costea T. Vlad O. C. Miclea L. C. Ganea C. Szöllősi J. Mocanu M. M. (2020). Alleviation of multidrug resistance by flavonoid and non-flavonoid compounds in breast, lung, colorectal and prostate cancer. Int. J. Mol. Sci. 21 (2), 401. 10.3390/ijms21020401 31936346 PMC7013436

[B31] Cruz-Zamora Y. Cruz-García F. Orozco-Arroyo G. Reyes-Santiago J. MartínezCastilla L. P. Wegier A. (2017). Polascontria (Cactaceae), a nothogenus that represents the gene flow between Escontria and Polaskia. Phytotaxa 295 (2), 18. 10.11646/phytotaxa.295.2.3

[B32] Dalla Via L. García-Argáez A. N. Martínez-Vázquez M. Grancara S. Martinis P. Toninello A. (2014). Mitochondrial permeability transition as target of anticancer drugs. Curr. Pharm. Des. 20 (2), 223–244. 10.2174/13816128113199990033 23701547

[B33] Daniloski D. D'Cunha N. M. Speer H. McKune A. J. Alexopoulos N. Panagiotakos D. B. (2022). Recent developments on Opuntia spp., their bioactive composition, nutritional values, and health effects. Food Biosci. 47 (21), 101665. Article 101665. 10.1016/j.fbio.2022.101665

[B34] Das G. Lim K. J. Tantengco O. A. G. Carag H. M. Gonçalves S. Romano A. (2021). Cactus: chemical, nutraceutical composition and potential bio-pharmacological properties. Phytotherapy Res. PTR 35 (3), 1248–1283. 10.1002/ptr.6889 33025610

[B35] da Silva Santos É. Braz de Oliveira A. J. de Fátima Pires da Silva Machado M. Mangolin C. A. Correia Gonçalves R. A. (2021). Cereus hildmannianus (K.) Schum. (Cactaceae): ethnomedical uses, phytochemistry and biological activities. J. Ethnopharmacol. 264, 113339. 10.1016/j.jep.2020.113339 32898627

[B36] da Silveira Agostini-Costa T. (2020). Bioactive compounds and health benefits of Pereskioideae and Cactoideae: a review. Food Chem. 327, 126961. 10.1016/j.foodchem.2020.126961 32422230

[B37] De Araujo F. F. Farias D. D. Neri-Numa I. A. Pastore G. M. (2021). Underutilized plants of the Cactaceae family: nutritional aspects and technological applications. Food Chem. 362 (17), 130196. Article 130196. 10.1016/j.foodchem.2021.130196 34091165

[B38] Dey P. Kundu A. Chakraborty H. J. Kar B. Choi W. S. Lee B. M. (2019). Therapeutic value of steroidal alkaloids in cancer: current trends and future perspectives. Int. J. cancer 145 (7), 1731–1744. 10.1002/ijc.31965 30387881 PMC6767045

[B39] Dhaouadi K. Raboudi F. Funez-Gomez L. Pamies D. Estevan C. Hamdaoui M. (2013). Polyphenolic extract of barbary-fig (Opuntia ficus-indica) syrup: RP–HPLC–ESI–MS analysis and determination of antioxidant, antimicrobial and cancer-cells cytotoxic potentials. Food Anal. Methods 6 (1), 45–53. 10.1007/s12161-012-9410-x

[B40] Djerassi C. Krakower G. W. Lemin A. J. Liu L. H. Mills J. S. Villotti R. (1958). The neutral constituents of the cactus Lophocereus Schottii. The structure of Lophenol--4α-Methyl-Δ7-cholesten-3β-ol--A link in sterol biogenesis1-3. J. Am. Chem. Soc. 80, 6284–6292. 10.1021/ja01556a031

[B42] Elansary H. O. Szopa A. Klimek-Szczykutowicz M. Jafernik K. Ekiert H. Mahmoud E. A. (2019). Mammillaria species-polyphenols studies and anti-cancer, anti-oxidant, and anti-bacterial activities. Mol. Basel, Switz. 25 (1), 131. 10.3390/molecules25010131 PMC6982789 31905725

[B43] El-Nashar H. A. S. Al-Azzawi M. A. Al-Kazzaz H. H. Alghanimi Y. K. Kocaebli S. M. Alhmammi M. (2024). HPLC-ESI/MS-MS metabolic profiling of white piraya fruit and cytotoxic potential against cervical cancer: comparative studies, synergistic effects, and molecular mechanistic approaches. J. Pharm. Biomed. analysis 244, 116121. 10.1016/j.jpba.2024.116121 38581932

[B45] Er H. M. Cheng E. H. Radhakrishnan A. K. (2007). Anti-proliferative and mutagenic activities of aqueous and methanol extracts of leaves from Pereskia bleo (Kunth) DC (Cactaceae). J. Ethnopharmacol. 113 (3), 448–456. 10.1016/j.jep.2007.06.026 17698306

[B46] Feugang J. M. Ye F. Zhang D. Y. Yu Y. Zhong M. Zhang S. (2010). Cactus pear extracts induce reactive oxygen species production and apoptosis in ovarian cancer cells. Nutr. cancer 62 (5), 692–699. 10.1080/01635581003605508 20574930

[B47] Franco-Molina M. Gomez-Flores R. Tamez-Guerra P. Tamez-Guerra R. Castillo-Leon L. Rodríguez-Padilla C. (2003). *In vitro* immunopotentiating properties and tumour cell toxicity induced by Lophophora williamsii (peyote) cactus methanolic extract. Phytotherapy Res. 17 (9), 1076–1081. 10.1002/ptr.1313 14595591

[B48] Franco-Molina M. A. Santana-Krímskaya S. E. Madrigal-de-León L. M. Coronado-Cerda E. E. Zárate-Triviño D. G. Hernández-Martínez S. P. (2021). Evaluation of the cytotoxic and immunogenic potential of temozolamide, panobinostat, and Lophophora williamsii extract against C6 glioma cells. EXCLI J. 20, 614–624. 10.17179/excli2020-3181 33883986 PMC8056056

[B50] Gandía-Herrero F. Escribano J. García-Carmona F. (2016). Biological activities of plant pigments betalains. Crit. Rev. food Sci. Nutr. 56 (6), 937–945. 10.1080/10408398.2012.740103 25118005

[B51] García-Cruz L. Valle-Guadarrama S. Guerra-Ramírez D. Martínez-Damian M. T. Zuleta-Prada H. (2022). Cultivation, quality attributes, postharvest behavior, bioactive compounds, and uses of Stenocereus: a review. Sci. Hortic. 304, 111336. 10.1016/j.scienta.2022.111336

[B52] Gomez-Flores R. Quintanilla-Licea R. Hernandez-Martinez H. C. Samaniego-Escamilla M. Tamez-Guerra P. Monreal-Cuevas E. (2019). Survival of lymphoma-bearing mice by Pachycereus marginatus cactus extracts and elucidation of bioactive compounds (Article). Nat. Product. Commun. 14 (5), 6. 10.1177/1934578x19845814

[B53] Guimarães D. De Castro D. de Oliveira F. L. Nogueira E. M. da Silva M. Teodoro A. J. (2017). Pitaya extracts induce growth inhibition and proapoptotic effects on human cell lines of breast cancer via downregulation of estrogen receptor gene expression. Oxidative Med. Cell. Longev. 2017, 7865073. 10.1155/2017/7865073 PMC5518493 28761624

[B54] Hahm S. W. Park J. Oh S. Y. Lee C. W. Park K. Y. Kim H. (2015). Anticancer properties of extracts from Opuntia humifusa against human cervical carcinoma cells. J. Med. Food 18 (1), 31–44. 10.1089/jmf.2013.3096 25379883

[B56] Harlev E. Nevo E. Solowey E. Bishayee A. (2013). Cancer preventive and curative attributes of plants of the Cactaceae family: a review. Planta Medica 79 (9), 713–722. 10.1055/s-0032-1328632 23702905

[B57] Heikal A. Abd El-Sadek M. E. Salama A. Taha H. S. (2021). Comparative study between *in vivo*- and *in vitro*-derived extracts of cactus (Opuntis ficus-indica L. Mill) against prostate and mammary cancer cell lines. Heliyon 7 (9), e08016. 10.1016/j.heliyon.2021.e08016 34622044 PMC8481975

[B58] Hernández-Martínez H. C. Gómez-Flores R. Tamez-Guerra P. Quintanilla-Licea R. Samaniego-Escamilla M. A. Monreal-Cuevas E. (2016). Antitumor activity of Pachycereus marginatus (DC.) Britton and Rose extracts against murine lymphoma L5178Y-R and skin melanoma B16F10 cells. J. Med. Plants Res. 10 (36), 635–639. 10.5897/JMPR2016.6156

[B59] Iqbal J. Abbasi B. A. Mahmood T. Kanwal S. Ali B. Shah S. A. (2017). Plant-derived anticancer agents: a green anticancer approach. Asian Pac. J. Trop. Biomed. 7 (12), 1129–1150. 10.1016/j.apjtb.2017.10.016

[B60] Izuegbuna O. Otunola G. Bradley G. (2019). Chemical composition, antioxidant, antiinflammatory, and cytotoxic activities of Opuntia stricta cladodes. PLoS ONE 14 (1), e0209682. 10.1371/journal.pone.0209682 30695064 PMC6350967

[B61] Jacomini D. Sinzker R. C. Mangolin C. A. Grande P. A. Nocchi S. R. Nakamura C. V. (2015). Lipid profile and antiproliferative activity of callus cultures of Cereus peruvianus Mill. Industrial Crops Prod. 69, 408–414. 10.1016/j.indcrop.2015.02.034

[B62] Jameel M. Rauf M. A. Khan M. T. Farooqi M. K. Alam M. A. Mashkoor F. (2023). Ingestion and effects of green synthesized cadmium sulphide nanoparticle on Spodoptera Litura as an insecticidal and their antimicrobial and anticancer activities. Pestic. Biochem. Physiol. 190, 105332. 10.1016/j.pestbp.2022.105332 36740336

[B63] Jayakumar R. Kanthimathi M. S. (2011). Inhibitory effects of fruit extracts on nitric oxide-induced proliferation in MCF-7 cells. Food Chem. 126 (3), 956–960. 10.1016/j.foodchem.2010.11.093

[B64] Joshi M. Prabhakar B. (2020). Phytoconstituents and pharmaco-therapeutic benefits of piraya: a wonder fruit. J. food Biochem. 44 (7), e13260. 10.1111/jfbc.13260 32378233

[B122] Khan T. Ali M. Khan A. Nisar P. Jan S. A. Afridi S. (2019). Anticancer plants: A review of the active phytochemicals, applications in animal models, and regulatory aspects. Biomolecules 10 (1), 47. 10.3390/biom10010047 31892257 PMC7022400

[B65] Keyvani-Ghamsari S. Khorsandi K. Gul A. (2020). Curcumin effect on cancer cells' multidrug resistance: an update. Phytotherapy Res. PTR 34 (10), 2534–2556. 10.1002/ptr.6703 32307747

[B66] Kim H. Choi H. K. Moon J. Y. Kim Y. S. Mosaddik A. Cho S. K. (2011). Comparative antioxidant and antiproliferative activities of red and white pitayas and their correlation with flavonoid and polyphenol content. J. food Sci. 76 (1), C38–C45. 10.1111/j.1750-3841.2010.01908.x 21535651

[B68] Kim J. Soh S. Y. Shin J. Cho C. W. Choi Y. H. Nam S. Y. (2015). Bioactives in cactus (Opuntia ficus-indica) stems possess potent antioxidant and pro-apoptotic activities through COX-2 involvement. J. Sci. food Agric. 95 (13), 2601–2606. 10.1002/jsfa.6968 25345579

[B69] Lee J. A. Jung B. G. Kim T. H. Lee S. G. Park Y. S. Lee B. J. (2013). Dietary feeding of Opuntia humifusa inhibits UVB radiation-induced carcinogenesis by reducing inflammation and proliferation in hairless mouse model. Photochem. Photobiol. 89 (5), 1208–1215. 10.1111/php.12113 23789636

[B71] Lefsih K. Giacomazza D. Passantino R. Costa M. A. Bulone D. Mangione M. R. (2018). Biochemical and biophysical characterization of water-soluble pectin from Opuntia ficus-indica and its potential cytotoxic activity. Phytochemistry 154, 47–55. 10.1016/j.phytochem.2018.06.015 30006087

[B72] Li W. Wu D. Wei B. Wang S. Sun H. Li X. (2014). Anti-tumor effect of cactus polysaccharides on lung squamous carcinoma cells (SK-MES-1). Afr. J. traditional, complementary, Altern. Med. AJTCAM 11 (5), 99–104. 10.4314/ajtcam.v11i5.16 PMC4202525 25395712

[B73] Liew S. Y. Stanbridge E. J. Yusoff K. Shafee N. (2012). Hypoxia affects cellular responses to plant extracts. J. Ethnopharmacol. 144 (2), 453–456. 10.1016/j.jep.2012.09.024 23022321

[B74] Lowry M. (2016). A synopsis of the genus Cleistocactus lemaire (cactaceae). Bradleya 34, 148–186. 10.25223/brad.n34.2016.a6

[B75] Luo H. Cai Y. Peng Z. Liu T. Yang S. (2014). Chemical composition and *in vitro* evaluation of the cytotoxic and antioxidant activities of supercritical carbon dioxide extracts of piraya (dragon fruit) peel. Chem. Central J. 8 (1), 1–7. 10.1186/1752-153X-8-1 PMC3880984 24386928

[B76] Madrigal-Santillán E. Portillo-Reyes J. Madrigal-Bujaidar E. Sánchez-Gutiérrez M. MercadoGonzalez P. E. Izquierdo-Vega J. A. (2022). Opuntia genus in human health: a comprehensive summary on its pharmacological, therapeutic and preventive properties. Part 1. Horticulturae 8, 88. 10.3390/horticulturae8020088 PMC9505094 36145735

[B78] Majolo F. de Oliveira Becker Delwing L. K. Marmitt D. J. BustamanteFilho I. C. Goettert M. I. (2019). Medicinal plants and bioactive natural compounds for cancer treatment: important advances for drug discovery. Phytochem. Lett. 31, 196–207. 10.1016/j.phytol.2019.04.003

[B79] Malek N. A. S. Sim K. Norhanom A. (2009b). Phytochemical and cytotoxic investigations of Pereskia grandifolia Haw. (Cactaceae) leaves. J. Biol. Sci. 9, 488–493. 10.3923/jbs.2009.488.493

[B80] Malek S. N. Shin S. K. Wahab N. A. Yaacob H. (2009a). Cytotoxic components of pereskia bleo (kunth) DC. (Cactaceae) leaves. Mol. Basel, Switz. 14 (5), 1713–1724. 10.3390/molecules14051713 PMC6254274 19471192

[B81] Malek S. N. A. Wahab N. A. Yaacob H. Shin S. K. Lai H. S. Serm L. G. (2008). Cytotoxic activity of Pereskia bleo (Cactaceae) against selected human cell lines. Int. J. Cancer Res. 4 (1), 20–27. 10.3923/ijcr.2008.20.27

[B82] Martínez E. Sandate-Flores L. Rodríguez-Rodríguez J. Rostro-Alanis M. Parra-Arroyo L. Antunes-Ricardo M. (2021). Underutilized Mexican plants: screening of antioxidant and antiproliferative properties of Mexican cactus fruit juices. Plants Basel, Switz. 10 (2), 368. 10.3390/plants10020368 PMC7918198 33672994

[B83] Martins M. Ribeiro M. H. Almeida C. M. M. (2023). Physicochemical, nutritional, and medicinal properties of *Opuntia ficus-indica* (L.) Mill. And its main agro-industrial use: a review. A Rev. Plants 12 (7), 1512. 10.3390/plants12071512 PMC10096643 37050137

[B84] Mina S. A. Melek F. R. Adeeb R. M. Haggag E. G. (2020). LC-ESI-MS/MS alkaloidal profiling and biological investigation of Cleistocactus winteri stems. Iran. J. Pharm. Res. IJPR 19 (2), 298–306. 10.22037/ijpr.2020.1101080 33224236 PMC7667565

[B85] Mohd-Salleh S. F. Ismail N. Wan-Ibrahim W. S. Tuan Ismail T. N. N. (2020a). Phytochemical screening and cytotoxic effects of crude extracts of pereskia bleo leaves. J. Herbs, Spices and Med. Plants 26, 291–302. 10.1080/10496475.2020.1729287

[B86] Mohd-Salleh S. F. Wan-Ibrahim W. S. Ismail N. (2020b). Pereskia bleo leaves extract induces cell death via cell cycle arrest and apoptosis in cervical cancer cells HeLa. Nutr. cancer 72 (5), 826–834. 10.1080/01635581.2019.1654530 31433251

[B87] Monteiro S. S. Almeida R. L. Santos N. C. Pereira E. M. Silva A. P. Oliveira H. M. L. (2023). New functional foods with cactus components: sustainable perspectives and future trends. Foods 12 (13), 2494. 10.3390/foods12132494 37444232 PMC10340198

[B88] Newman D. J. Cragg G. M. (2020). Natural products as sources of new drugs over the nearly four decades from 01/1981 to 09/2019. J. Nat. Prod. 83, 770–803. 10.1021/acs.jnatprod.9b01285 32162523

[B89] Orozco-Barocio A. Paniagua-Domínguez B. L. Benítez-Saldaña P. A. Flores-Torales E. Velázquez-Magaña S. Nava H. J. (2013). Cytotoxic effect of the ethanolic extract of Lophocereus schottii: a mexican medicinal plant. Afr. J. traditional, complementary, Altern. Med. AJTCAM 10 (3), 397–404. PMC3777577 24146465

[B90] Orozco-Barocio A. Robles-Rodríguez B. S. Camacho-Corona M. Méndez-López L. F. Godínez-Rubí M. Peregrina-Sandoval J. (2022). *In vitro* anticancer activity of the polar fraction from the Lophocereus schottii ethanolic extract. Front. Pharmacol. 13, 820381. 10.3389/fphar.2022.820381 35444555 PMC9014087

[B91] Ortiz-González A. González-Pérez P. P. Cárdenas-García M. Hernández-Linares M. G. (2022). *In silico* prediction on the PI3K/AKT/mTOR pathway of the antiproliferative effect of O. Joconostle in breast cancer models. Cancer Inf. 21, 11769351221087028. 10.1177/11769351221087028 PMC8958723 35356703

[B92] Pasko P. Galanty A. Zagrodzki P. Ku Y. G. Luksirikul P. Weisz M. (2021a). Bioactivity and cytotoxicity of different species of piraya fruits – a comparative study with advanced chemometric analysis. Food Biosci. 40 (8), 100888. Article 100888. 10.1016/j.fbio.2021.100888

[B93] Pasko P. Galanty A. Zagrodzki P. Luksirikul P. Barasch D. Nemirovski A. (2021b). Dragon fruits as a reservoir of natural polyphenolics with chemopreventive properties. Mol. Basel, Switz. 26 (8), 2158. 10.3390/molecules26082158 PMC8070077 33918584

[B94] Pimienta-Barrios E. Nobel P. S. (1994). Pitaya (Stenocereus spp, cactaceae) – an ancient and modern fruit crop of Mexico. Econ. Bot. 48 (1), 76–83. 10.1007/Bf02901385

[B95] Pinto N. C. C. dos Santos R. C. Cunha Machado D. Rodrigues F. J. de Souza Fagundes E. M. Antinarelli L. M. R. (2012). Cytotoxic and antioxidant activity of Pereskia aculeata miller. PhOL – Pharmacol. Online 3, 63–69.

[B96] Polini G. Camaqui M. A. (2018). Plantas Medicinales de Bolivia. Tiquipalla, Potosi: CreateSpace Independent Publishing Platform, 7–8. ISBN-10 : 1985132419, ISBN-13 ‏: 978-1985132412.

[B97] Quintanilla-Licea R. Gomez-Flores R. Samaniego-Escamilla M. A. Hernández-Martínez H. C. Tamez-Guerra P. Morado-Castillo R. (2016). Cytotoxic effect of methanol extracts and partitions of two Mexican desert plants against the murine lymphoma L5178Y-R. Am. J. Plant Sci. 7, 1521–1530. 10.4236/ajps.2016.711143

[B98] Ramírez-Rodríguez Y. Martínez-Huélamo M. Pedraza-Chaverri J. Ramírez V. Martínez-Tagüeña N. Trujillo J. (2020). Ethnobotanical, nutritional and medicinal properties of Mexican dryland Cactaceae Fruits: recent findings and research opportunities. Food Chem. 312, 126073. 10.1016/j.foodchem.2019.126073 31901824

[B99] Ríos-León K. Fuertes-Ruiton C. Arroyo J. Ruiz J. (2017). Chemoprotective effect of the alkaloid extract of Melocactus bellavistensis against colon cancer induced in rats using 1,2-dimethylhydrazine. Rev. Peru. Med. Exp. salud publica 34 (1), 70–75. 10.17843/rpmesp.2017.341.2768 28538848

[B100] Rizeq B. Gupta I. Ilesanmi J. AlSafran M. Rahman M. M. Ouhtit A. (2020). The power of phytochemicals combination in cancer chemoprevention. J. Cancer 11, 4521–4533. 10.7150/jca.34374 32489469 PMC7255361

[B101] Salam H. S. Tawfik M. M. Elnagar M. R. Mohammed H. A. Zarka M. A. Awad N. S. (2022). Potential apoptotic activities of Hylocereus undatus peel and pulp extracts in MCF-7 and Caco-2 cancer cell lines. Plants 11 (17), 2192. 10.3390/plants11172192 36079573 PMC9459728

[B102] Salazar J. R. Martínez-Vazquez M. Cespedes C. L. Ramírez-Apan T. Nieto-Camacho A. Rodríguez-Silverio J. (2011). Anti-inflammatory and cytotoxic activities of chichipegenin, peniocerol, and macdougallin isolated from Myrtillocactus geometrizans (Mart. ex Pfeiff.) Con. J. Biosci. 66 (1-2), 0024–0030. 10.5560/znc.2011.66c0024 21476433

[B103] Sandate-Flores L. Romero-Esquivel E. Rodríguez-Rodríguez J. Rostro-Alanis M. Melchor-Martínez E. M. Castillo-Zacarías C. (2020). Functional attributes and anticancer potentialities of chico (Pachycereus weberi) and jiotilla (Escontria chiotilla) fruits extract. Plants Basel, Switz. 9 (11), 1623. 10.3390/plants9111623 PMC7700655 33266445

[B104] Santos Díaz M. D. S. Barba de la Rosa A. P. Héliès-Toussaint C. Guéraud F. Nègre-Salvayre A. (2017). Opuntia spp.: characterization and benefits in chronic diseases. Oxidative Med. Cell. Longev. 2017, 8634249. 10.1155/2017/8634249 PMC5401751 28491239

[B105] Serra A. T. Poejo J. Matias A. A. Bronze M. R. Duarte C. M. M. (2013). Evaluation of Opuntia spp. derived products as antiproliferative agents in human colon cancer cell line (HT29). Food Res. Int. 54, 892–901. 10.1016/j.foodres.2013.08.043

[B106] Sharif K. M. Rahman M. M. Zaidul I. S. M. Jannatul A. Akanda M. J. H. Mohamed A. (2013). Pharmacological relevance of primitive leafy cactuses Pereskia. (Review). Res. J. Biotechnol. 8 (12), 134–142.

[B107] Shetty A. A. Rana M. K. Preetham S. P. (2012). Cactus: a medicinal food. J. Food Sci. Technol. 49, 530–536. 10.1007/s13197-011-0462-5 24082263 PMC3550841

[B108] Shoukat R. Cappai M. Pia G. Pilia L. (2023). An updated review: Opuntia ficus indica (OFI) chemistry and its diverse applications. Appl. Sci. 13 (13), 7724. 10.3390/app13137724

[B109] Shyamalagowri S. Charles P. Manjunathan J. Kamaraj M. Anitha R. Pugazhendhi A. (2022). *In vitro* anticancer activity of silver nanoparticles phyto-fabricated by Hylocereus undatus peel extracts on human liver carcinoma (HepG2) cell lines. Process Biochem. 116, 17–25. 10.1016/j.procbio.2022.02.022

[B110] Siew Y. Y. Yew H. C. Neo S. Y. Seow S. V. Lew S. M. Lim S. W. (2019). Evaluation of anti-proliferative activity of medicinal plants used in Asian Traditional Medicine to treat cancer. J. Ethnopharmacol. 235, 75–87. 10.1016/j.jep.2018.12.040 30599223

[B111] Sreekanth D. Arunasree M. K. Roy K. R. Chandramohan Reddy T. Reddy G. V. Reddanna P. (2007). Betanin a betacyanin pigment purified from fruits of Opuntia ficus-indica induces apoptosis in human chronic myeloid leukemia Cell line-K562. Phytomedicine Int. J. phytotherapy Phytopharm. 14 (11), 739–746. 10.1016/j.phymed.2007.03.017 17482444

[B112] Tan M. L. Sulaiman S. F. Najimuddin N. Samian M. R. Muhammad T. S. (2005). Methanolic extract of Pereskia bleo (Kunth) DC. (Cactaceae) induces apoptosis in breast carcinoma, T47-D cell line. J. Ethnopharmacol. 96 (1-2), 287–294. 10.1016/j.jep.2004.09.025 15588681

[B113] Vencioneck Dutra J. C. Moisés Ferreira J. Costalonga Pereira P. R. Ben-Hur de Oliveira J. Vitorino Gervásio S. Bernardes Xavier M. (2018). Cereus jamacaru D.C. hydroalcoholic extract promotes anti-cytotoxic and antitumor activity. Pharm. Basel, Switz. 11 (4), 130. 10.3390/ph11040130 PMC6316405 30477180

[B114] Verona-Ruiz A. Urcia-Cerna J. Paucar-Menacho L. M. (2020). Pitahaya (Hylocereus spp.): culture, physicochemical characteristics, nutritional composition, and bioactive compounds. Sci. Agropecu. 11 (3), 439–453. 10.17268/sci.agropecu.2020.03.16

[B115] Wang J. Rani N. Jakhar S. Redhu R. Kumar S. Kumar S. (2023). Opuntia ficus-indica (L.) Mill. - anticancer properties and phytochemicals: current trends and future perspectives. Front. plant Sci. 14, 1236123. 10.3389/fpls.2023.1236123 37860248 PMC10582960

[B117] World Health Organization (WHO) (2024). Cancer today. Available at: https://gco.iarc.fr/today/en (Accessed august 27, 2024).

[B118] Wu L. Hsu H.-W. Chen Y.-C. Chiu C.-C. Lin Y.-I. Ho J. A. (2006). Antioxidant and antiproliferative activities of red piraya. Food Chem. 95 (2), 319–327. 10.1016/J.FOODCHEM.2005.01.002

[B120] Zhi-Yu Z. Xiao-Tao S. Hao-Peng Z. Xue-li S. Zhang Y. Jing-wei Z. (2011). Inhibitory and apoptosis-inducing effects of Lophophora williamsii extracts on HeLa cells. J. Med. Plants Res. 5 (8), 1305–1312.

[B121] Zou D. M. Brewer M. Garcia F. Feugang J. M. Wang J. Zang R. (2005). Cactus pear: a natural product in cancer chemoprevention. Nutrition Journal. 4, 25. 10.1186/1475-2891-4-25 16150152 PMC1242252

